# Cellular requirements for PIN polar cargo clustering in *Arabidopsis thaliana*

**DOI:** 10.1111/nph.16887

**Published:** 2020-09-18

**Authors:** Hongjiang Li, Daniel von Wangenheim, Xixi Zhang, Shutang Tan, Nasser Darwish‐Miranda, Satoshi Naramoto, Krzysztof Wabnik, Riet De Rycke, Walter A. Kaufmann, Daniel Gütl, Ricardo Tejos, Peter Grones, Meiyu Ke, Xu Chen, Jan Dettmer, Jiří Friml

**Affiliations:** ^1^ Institute of Science and Technology Austria (IST Austria) Klosterneuburg 3400 Austria; ^2^ Department of Plant Systems Biology, VIB and Department of Plant Biotechnology and Bioinformatics Ghent University Ghent 9052 Belgium; ^3^ Centre for Plant Integrative Biology School of Biosciences University of Nottingham Loughborough LE12 5RD UK; ^4^ Department of Applied Genetics and Cell Biology University of Natural Resources and Life Sciences (BOKU) Vienna 1190 Austria; ^5^ Graduate School of Life Sciences Tohoku University Sendai 980‐8577 Japan; ^6^ Department of Plant Biotechnology and Bioinformatics Ghent University Ghent 9052 Belgium; ^7^ VIB Center for Plant Systems Biology Ghent 9052 Belgium; ^8^ Expertise Centre for Transmission Electron Microscopy and VIB BioImaging Core Ghent University Ghent 9052 Belgium; ^9^ Departamento de Biología Facultad de Ciencias Centro de Biología Molecular Vegetal Universidad de Chile Santiago 7800003 Chile; ^10^ Haixia Institute of Science and Technology Fujian Agriculture and Forestry University Fuzhou 350002 China

**Keywords:** *Arabidopsis*, auxin, cell wall, cluster, cytoskeleton, PIN, PIP5K, polarity

## Abstract

- Cell and tissue polarization is fundamental for plant growth and morphogenesis. The polar, cellular localization of *Arabidopsis* PIN‐FORMED (PIN) proteins is crucial for their function in directional auxin transport. The clustering of PIN polar cargoes within the plasma membrane has been proposed to be important for the maintenance of their polar distribution. However, the more detailed features of PIN clusters and the cellular requirements of cargo clustering remain unclear.
- Here, we characterized PIN clusters in detail by means of multiple advanced microscopy and quantification methods, such as 3D quantitative imaging or freeze‐fracture replica labeling. The size and aggregation types of PIN clusters were determined by electron microscopy at the nanometer level at different polar domains and at different developmental stages, revealing a strong preference for clustering at the polar domains.
- Pharmacological and genetic studies revealed that PIN clusters depend on phosphoinositol pathways, cytoskeletal structures and specific cell‐wall components as well as connections between the cell wall and the plasma membrane.
- This study identifies the role of different cellular processes and structures in polar cargo clustering and provides initial mechanistic insight into the maintenance of polarity in plants and other systems.

## Introduction

Auxin is a fundamental plant hormone that plays crucial roles in a plethora of developmental processes (Mockaitis & Estelle, 2008; Grones & Friml, 2015). The key mechanism in auxin action is its directional (polar) transport between cells for its differential distribution in plant tissues. This process underlies a plethora of developmental processes, such as embryonic axis establishment, root and shoot tropisma, meristem activities, and root and shoot organogenesis (Adamowski & Friml, 2015). Polarly localized members of the plant‐specific family of PIN‐FORMED (PIN) auxin transporters regulate both the rate and the directionality of this auxin transport that is essential to connect polarities at the individual cell level and the tissue and organ levels (Wiśniewska *et al*., 2006; Sauer *et al*., 2006a; Glanc *et al*., 2018; Skokan *et al*., 2019; Mazur *et al*., 2020; Zhang *et al*., 2020). In animal, cell polarity is regulated through several conserved factors (Crumbs, Scribble and PAR) that are absent in known plant genomes (Geldner, 2009; Kania *et al*., 2014), and tight junctions separating the polar domains between neighboring epithelial cells (Nelson & Beitel, 2009). Instead of tight junctions, plants possess a cell wall, a crucial cellular component that provides structural integrity to plant tissues and controls cellular growth and architecture (Wolf *et al*., 2012a). Mechanical strains exerted on the cell wall are transmitted toward the plasma membrane (PM) through still undiscovered connections. Both PIN polarity and endocytic trafficking can be affected by mechanical or osmotic stresses (Nakayama *et al*., 2012; Zwiewka *et al*., 2015) and PIN internalization is also regulated by the cytoskeleton‐linked Rho‐like GTPase (Craddock & Yang, 2012).

How can polarity of the polar cargoes be maintained within the rather fluid PM in plant cells in the absence of diffusion barriers? An experimental and computational simulation revealed that within the PM polar domains, PIN proteins are recruited into nonmobile signal aggregates, called clusters, suggesting that PIN clusters may play a role in polar auxin transport. This phenomenon might be critical for polarity maintenance together with endosome‐guided cargo recycling, super‐polar targeting of PIN proteins to the center of polar domains, and PIN protein retrieval at the lateral cell side by clathrin‐dependent endocytosis (Kleine‐Vehn *et al*., 2008, Kleine‐Vehn *et al*., 2011; Glanc *et al*., 2019; Narasimhan *et al*., 2020). Clustering, which has been reported to regulate polarity in *Schizosaccharomyces pombe* (fission yeast), *Saccharomyces cerevisiae* (baker’s yeast) and *Caenorhabditis elegans* cells (Dodgson *et al*., 2013), might be a universal way to regulate protein polarity, and its mechanism may well be conserved. However, the characteristics of PIN clusters and the cellular and genetic factors that regulate clustering of polar cargoes remain unclear.

Genetic and pharmacological interference with the deposition of cellulose, which is the major component of the cell wall, and mechanical disruption of the cell wall increased lateral diffusion and defects in the polar distribution of PIN proteins (Feraru *et al*., 2011). A connection between microtubule arrangements, the cell wall and PIN1 localization has also been suggested in the shoot apical meristem (Heisler *et al*., 2010). In addition, the cell wall has been shown to play a crucial role in immobilizing PM proteins and the cellulose deposition pattern in the cell wall to affect the trajectory and speed of PM protein diffusion (Martinière *et al*., 2012). Although both cell wall and polar cargo clustering are important for lateral diffusion and polarity, their exact roles and mutual relationship remain unclear. Here, we dissected the cellular characteristics of PIN clusters by using different microscopy methods and identified the cellular factors involved in polar cargo clustering, such as lipid kinase pathways, the cytoskeleton, cell wall and cell wall–PM connection.

## Materials and Methods

### Plant growth conditions

*Arabidopsis thaliana* (L.) Heyhn. seeds were sterilized by chlorine gas or 75% (v/v) ethanol and sown on plates containing half‐strength Murashige and Skoog (½MS) medium (pH 5.7) with 1% (w/v) sucrose and 0.8% (w/v) agar. After stratification at 4°C for 2 d, plates with seeds were transferred to a growth room at 22°C under a 16 h : 8 h, light : dark photoperiod. The seedlings were grown vertically for 3 d. The transgenic reporter lines were described previously: *PIN2::PIN2‐GFP* in *eir1‐4* (Baster *et al*., 2012). *PIN2::PIN2‐Venus* (Leitner *et al*., 2012), *PIN1::PIN1‐GFP* (Benková *et al*., 2003), *35S::GFP‐PIP2a* (Cutler *et al*., 2000), *ndr1‐1* mutant, *ndr1‐1*/*NDR1::T7‐NDR1* and *ndr1‐1*/*35S:HA‐NDR1* (Coppinger *et al*., 2004; Day *et al*., 2006), *XVE>>YFP‐PIP5K1* (Tejos *et al*., 2014), *pip5k1 pip5k2* (Tejos *et al*., 2014), *pROP6>>GFP‐rop6* CA (Poraty‐Gavra *et al*., 2013), *35S::P30‐GFP* (Kim *et al*., 2005), *cesa3^je5^* (Desprez *et al*., 2007) and *cesa6^prc1‐1^* (Fagard *et al*., 2000; Desprez *et al*., 2007). The *cesa3; PIN2‐GFP* line was *cesa3^je5^* crossed with *PIN2::PIN2‐GFP* and the *cesa6; PIN2‐GFP* line was *cesa6^prc1‐1^* crossed with *PIN2::PIN2‐GFP*. For the generation of *pREM1.2::GFP‐REM1.2* constructs, a 1.5 kb promoter and full‐length REM1.2 was cloned into the Gateway pDNORP4P1R and pDONR221 vectors, respectively, and subsequently cloned into pB7m24GW2 vector using Gateway cloning technology (www.invitrogen.com). The resultant constructs were introduced in Columbia (Col‐0) or individual *rem* mutants by *Agrobacterium*‐mediated genetic transformation.

### Confocal microscopy

Three days after germination, the *Arabidopsis* seedlings were mounted in liquid ½MS under a cover glass or under a small piece of growth medium agar in a chamber with a cover glass bottom. In most cases, the meristematic root zone was imaged, but other regions were imaged in some experiments as indicated (Johnson *et al*., 2020). Confocal images were obtained with a Zeiss LSM 700 using a ×100/NA 1.46 oil objective lens. Green fluorescent protein (GFP) fluorescence was excited at 488 nm (laser power 10%), and emission was collected 493 nm. Data for Supporting Information Videos S1 and S2 were taken with a spinning disk microscope (Andor Spinning Disc System; Andor Technology, Belfast, UK) with an inverted observer (Zeiss) and a ×100/NA 1.4 oil objective. The reconstruction of confocal 3D images and videos were processed in Imaris (v.7.7.4).

### Vibratome root cutting

The 3‐d‐old seedlings were imbedded into 5% (w/v) low‐melting analytical agarose (Promega). The agar with roots was cut into cubes and glued onto the blade of Vibratome VT 1200S (Leica Microsystems, Wetzlar, Germany). Sections of 200–400 µm thickness were cut and placed on slides mounted with water for immediate observation under the confocal microscope.

### Postembedding immunogold transmission electron microscopy

Root tips of 3‐d‐old seedlings of wild type or chemically treated *Arabidopsis* seedlings were excised, immersed in 20% (w/v) BSA, and frozen immediately in a high‐pressure freezer (EM PACT; Leica Microsystems). Freeze substitution was carried out in an EM AFS_2_ (Leica Microsystems). Cells were freeze‐substituted in dry acetone with 0.1% (v/v) glutaraldehyde for 4 d as follows: −90°C for 24 h, 2°C h^–1^ increase for 15 h, −60°C for 16 h, 2°C h^–1^ increase for 15 h, and −30°C for 8 h. At −30°C, the carriers were rinsed three times with acetone for 20 min each time. Samples were then slowly warmed to 4°C, stepwise infiltrated over 3 d at 4°C in hard‐grade LR‐white resin (London Resin) and embedded in capsules. Polymerization was done in an EM AFS (Leica Microsystems) with UV illumination over 6 d, starting at 0°C and ending at 37°C. Ultrathin sections of gold interference color were cut with an ultramicrotome (EM UC6; Leica Microsystems) and collected on Formvar‐coated copper mesh grids. All immunolabeling steps were done in a humid chamber at room temperature. Grids were floated upside down on 25 µl aliquots of blocking solution (5% (w/v) BSA, 1% (w/v) fish skin gelatin (FSG) in PBS) for 20 min, followed by washing five times for 5 min each wash (1% (w/v) BSA in PBS). Grids were incubated in a 1 : 300 dilution (1% (w/v) BSA in PBS) of biotin‐conjugated goat anti‐GFP primary antibodies (Rockland 600‐106‐215) for 120 min, followed by washing five times for 5 min each wash (0.1% (w/v) BSA in PBS). The grids were then incubated with a 1 : 10 000 dilution of unconjugated rabbit anti‐biotin (Rockland 100‐4198) bridging antibodies for 30 min, followed by washing five times for 5 min (0.1% (w/v) BSA in PBS). After a final incubation with protein A/10‐nm gold (PAG_10nm_; Cell Biology, Utrecht University, the Netherlands), grids were sequentially washed twice for 5 min each time with 0.1% (w/v) BSA in PBS, PBS and double‐distilled water. Control experiments consisted of treating sections with bridging antibodies and/or PAG_10nm_ alone. Sections were post‐stained in an automatic EM AC20 contrasting system (Leica Microsystems) for 30 min in uranyl acetate at 20°C and for 7 min in the lead stain at 20°C. Grids were viewed with a transmission electron microscope (JEM1010; Jeol, Tokyo, Japan) operating at 80 kV with the Image Plate Technology from Ditabis (Pforzheim, Germany). For each sample, cluster numbers and distribution were calculated from the analysis of at least 19 images, at least five seedlings and 15 cells that had been analyzed.

### SDS‐digested freeze‐fracture replica labeling

Sodium dodecyl sulfate‐digested freeze‐fracture replica labeling (SDS‐FRL) was applied with some modifications from the method described for mammalian tissue samples (Kaufmann *et al*., 2010; Möbius *et al*., 2010). Details of the modified method are provided in Methods S1.

### Sampling and analysis of SDS‐FRL data

Four to seven replicas were used for quantification of immunolabeling per area of interest that were apical (shoot apex‐facing), lateral and basal (root apex‐facing) domains of *PIN2‐Venus* and *PIP2a‐GFP* epidermal cells. Within these areas, profiles were selected at random and electron micrographs were taken at a magnification of ×39 000 to ×93 000. The magnification was verified by a calibration grid. Quantification was done either manually with the item ce software (Olympus Soft Imaging Solutions, Münster, Germany) or semi‐automatically using fiji and matlab. The semi‐automatic method was done as follows, in fiji: black shadows were manually overdrawn with white, and then a threshold was applied manually to highlight gold particles only. The plugin ‘Analyze Particles’ was applied. The obtained list of coordinates was imported into matlab and particles were assigned to groups based on their maximal distance of 55 nm. Data were expressed as mean ± SD. To compare the density of immunoparticles in different domains and genetic lines, a Mann–Whitney *U*‐test (*P* = 0.05) test was applied. Statistical analysis was carried out in prism (GraphPad, La Jolla, CA, USA). At least 25 cells were analyzed.

### Quantification of clusters from confocal microscopy

The clusters are distributed in cell membranes, generally perpendicular to the optical axis of the microscope. Hence, the quality of the images is limited by the axial (*Z*) resolution of the objective lens used. For confocal microscopy, a ×100/1.46 oil objective lens was used with a Zeiss LSM700 confocal microscope. The obtained pixel sizes were 0.089/0.089/0.313 µm (*x*/*y*/*z*). The axial asymmetry was ignored, assuming that elongations in *Z* are artificial. As the very small volumes made segmentation problematic, a blob detection algorithm was implemented to find the clusters. For computational convenience, the Maximum Intensity Projections (MIPs) of the axial image sections were used to identify the local maxima and to classify them according to their size. First, the brightest pixels (local maxima) were localized. Each of these local maxima is the absolute maximum of its local 3 × 3 group of pixels. In other words, the eight nearest neighbors around it receive dimmer intensities. The next step is to estimate the corresponding cluster size for each maximum. This is done by dilating the local domains around them successively, and testing if the pixels in these larger domains start to become brighter again. The considered domains have initially 3 × 3 pixels (the considered pixel and its nearest neighbors), then 5 × 5 pixels to incorporate the second neighbors, then 7 × 7 pixels, etc. The area across which no brighter pixels are found defines the estimated size of the clusters. For a radius of *n* pixels, a 2*n* + 1 × 2*n* + 1 pixel domain has to be considered. To analyze large sets of cell membranes, we developed a software program in Matlab (MathWorks, Natick, MA, USA). A batch series involving the steps described above was executed on a series of regions of interest (ROIs) selected interactively by the user. These ROIs might have different sizes, but the surface concentrations are given. We expect to find some correlation of the surface cluster densities for cells of the same type, and some significant difference from one cell type to another.

At small sizes, the cluster detection was not reliable. A simple explanation might be that the numbers were not consistent because the observed structures were not the clusters of interest, the size of which we did not know in advance. At the large extreme, other structures were found instead, such as overlapped sections of the same membrane. Between these boundaries, clusters ranging from 0.44 µm (5 pixels) to 0.80 µm (9 pixels) at the confocal microscope were selected. We considered that any thresholding would bias the analyses, and for this reason, we excluded the use of the actual intensity values for blob detection. The way to do it was to compare intensity differences between neighbors, but not particular intensity values. In this way, the brightest spot of a 9 × 9‐pixel domain will be classified as an 8‐pixel‐diameter cluster, regardless of whether the whole group is brighter or dimmer. The only condition is that the intensities decrease consistently from the maximum away. This was very robust in general, but some false positives might arise if a bright pixel appears in a very dim area, such as the border of another cell in a dark area. For this reason, the projections were checked manually and some cases were discarded.

### Immunolocalization

Three‐ to 4‐d‐old seedlings were immunolocalized using an *in situ* pro robot (Intavis, Cologne, Germany) according to the described protocol (Sauer *et al*., 2006b). The primary antibodies were rabbit anti‐PIN2 (Abas *et al*., 2006) 1 : 1000 and mouse anti‐GFP (Sigma‐Aldrich) 1 : 600, and the secondary antibodies were Cy3 anti‐rabbit (Sigma‐Aldrich) 1 : 600 and Alexa488 anti‐mouse (Invitrogen) 1 : 600.

### Lipid‐protein blot overlay assay

The lipid‐protein binding assay was performed as previously described (Tan *et al*., 2020b). In brief, the recombinant His‐PIN2HL was expressed and purified from *Escherichia coli*. PIP strips (P‐6001, Echelon Bioscience, Salt Lake City, UT, USA) membrane was blocked in the blocking buffer with 3% BSA in 1× TBST for 1 h. Purified His‐PIN2HL (20 µl in 10 ml 1× TBST) was incubated with the membrane for 2 h. The membrane was washed three times for 5 min with 1× TBST, and then incubated with anti‐His HRP‐conjugated antibody (dilution 1 : 4000; Agrisera, Vännäs, Sweden) for 2 h at room temperature. After washing three times for 5 min with 1× TBST, the bound protein was detected using SuperSignal western detection reagents (Thermo Fisher Scientific, Waltham, MA, USA) in an Amersham 600RGB molecular imaging system (GE Healthcare, Little Chalfont, UK).

### Auxin transport assay

The auxin transport assay was performed in etiolated hypocotyls as previously described (Lewis & Muday, 2009; Tan *et al*., 2020a). First, 6‐d‐old etiolated seedlings were transferred to the MS medium plates. For 1‐N‐naphthylphthalamic acid (NPA) treatment, 6‐d‐old etiolated Col‐0 seedlings were transferred to the MS medium plates supplemented with 5 µM NPA. MS medium/1.25% agar droplets with 500 µM ^3^H‐IAA were prepared and were placed on the upper part of decapitated hypocotyls of 6‐d‐old seedlings, one droplet per hypocotyl. Fifteen hypocotyls were regarded as one replicate, with three replicates per genotype. After incubation in the dark for 6 h, upperparts with ^3^H‐IAA droplet and roots of hypocotyls were cut off, and the rest of the hypocotyls were collected and frozen in liquid nitrogen, and then homogenized in the 1 ml scintillation solution. After incubation in the scintillation solution overnight, the samples were evaluated with a scintillation counter (Hidex 300XL). The sample with only 1 ml scintillation solution was also measured as a background control.

### Chemical treatments

*PIN2‐Venus* seedlings were treated with 30 µM wortmannin (WM) for 2 h, 0.8% (v/v) 1‐butanol or 2‐butanol for 3 h (Li & Xue, 2007), 10 µM U‐73343 or U‐73122 for 2 h (Chu *et al*., 2016), 40 µM oryzalin for 1–3 h (with similar results), 20 µM latrunculin B for 2 h, 0.1% (w/v) macerozyme for 5 min, 5% (w/v) cellulase for 5 min, 5 nM isoxaben for 3 h, 0.1% (v/v) driselase for 15 min, and 0.5 M mannitol (Feraru *et al*., 2011) and 100 µM AlCl_3_ for 1–3 h (Yang *et al*., 2014). At least 10 roots, five cells per root were analyzed for each treatment. For the observation of the localization of the estrogen receptor‐based chemical‐inducible *XVE>PIP5K1‐YFP* expression, 3‐d‐old seedlings were treated with 2.5 µM estradiol in liquid plant growth medium overnight before observation. For the epigallocatechin gallate (EGCG) treatment plants were sprayed with 50 µM EGCG or H_2_O as control. SDS‐FRL was performed 24 h after treatment.

### Accession numbers

Sequence data from this article can be found in the *Arabidopsis* Genome Initiative or GenBank/EMBL databases under the following accession numbers: PIN1, At1g73590; PIN2, At5g57090; PIP2a, At3G53420; PIP5K1, At1g21980; PIP5K2, At1g77740; KTN1, At1g80350; TOR1/SPR2, At4g27060; TOR2/TUA4, At1g04820; CESA3, At5g05170; CESA6, At5g64740; and NDR1, At3g20600.

## Results

### Clustering of polar cargos observed by confocal microscopy

**Fig. 1:**
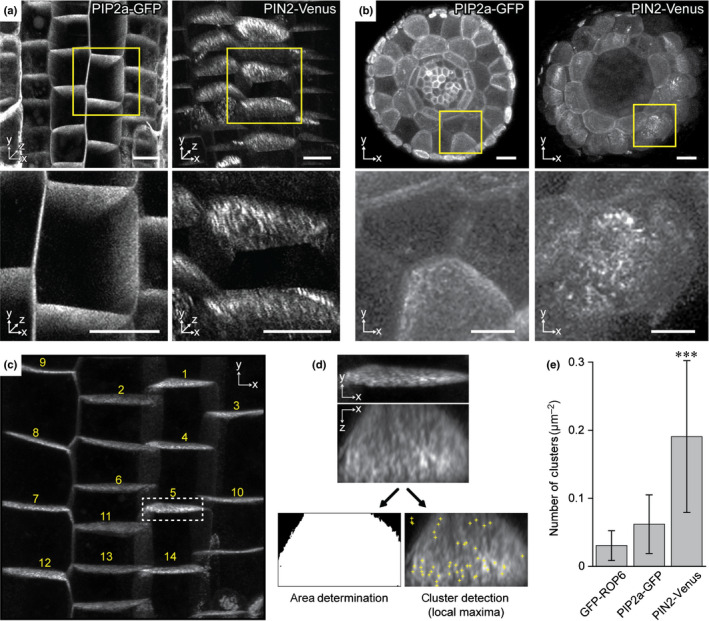
Visualization and quantification of *Arabidopsis* PIN clusters by confocal microscopy. (a) Three‐dimensional view of clustering of the nonpolar marker PIP2a‐GFP (left) and the polarly localized PIN2‐Venus (right). Lower panel shows enlargements of the yellow boxes from upper panel. Bars, 10 µm. (b) Vibratome‐cut cross‐section of the root tip meristematic zone of PIP2a‐GFP (left) and PIN2‐Venus (left). Bars, 10 µm. Lower panel shows enlargements of the yellow boxes from upper panel. Bars, 10 µm. (c) Cluster quantification in the matlab program. Cell membranes are marked and numbered. (d) Enlargement of cell membrane number 5 (white dashed box). Each cropped substack was projected (maximum intensity) along the *x*/*z* direction. To identify clusters local maxima were found and grouped using matlab (see detailed description in the Materials and Methods section). Brightness thresholding reveals the area to calculate the actual density of clusters per area. (e) Quantitative analysis of the number of clusters with diameter sizes from 0.45 to 0.8 µm per µm^2^ in GFP‐ROP6 (0.03 ± 0.02), PIP2a‐GFP (0.06 ± 0.04) and PIN2‐Venus (0.19 ± 0.11). Values are means ± SD from at least 10 cells. Mann–Whitney *U*‐test: ***, *P* < 0.001 vs either ROP6 or PIP2a.

In *A. thaliana*, the auxin transporters PIN1 and PIN2 are polarly localized proteins that form clusters at the PM (Kleine‐Vehn *et al*., 2011). We examined *PIN2‐Venus* clusters (Leitner *et al*., 2012) in three dimensions and compared them to the nonpolar membrane marker *PIP2a‐GFP* (Plasma Membrane Intrinsic Protein 2a) (Cutler *et al*., 2000) as a control (Fig. 1a,b,e). We selected the nonpolar PM aquaporin PIP2a based on its localization and functional differences from PINs: polarly localized PINs are located in different membrane microdomains from PIP2a and PIN directionally transports auxin, while PIP2a is internalized upon exposure to salt stress conditions (Chevalier & Chaumont, 2014). Video S1 shows the distribution of PIN2‐GFP in a 3D rotation of a typical image stack. Live‐cell imaging showed that the *PIN2‐GFP* clustering appears to be stable over 13 min while the root tip grows through the field of view (Video S2). Due to the limited axial (*Z*) resolution of the objective lens in light microscopes, clusters appear longer in the *z*‐direction. To show that this is a purely optical artefact and not caused by the shape of clusters, we visualized clusters of PIN2 and PIP2a in vibratome‐cut cross‐sections of the meristematic zone of the root tip (Fig. 1b). However, all quantifications of clusters were done in living samples and showed pronounced clusters in *PIN2‐Venus* when compared to the nonpolar marker *PIP2a‐GFP* or *ROP6‐GFP* (Fig. 1e). To quantify the clusters from *Z*‐stack confocal images, we developed software in the Matlab environment (Fig. 1c,d). Using this software, we quantified cluster densities in different zones and at various developmental stages and found similar PIN2 cluster densities throughout the root cap, transition and meristem zones (Fig. S1). Clustering of the polar‐localized PIN2 appeared to be specific to these polar PM proteins because the nonpolar PIP2a marker does not form comparable clusters.

### Clustering at different polar domains observed by post‐embedding immunogold electron microscopy

**Fig. 2:**
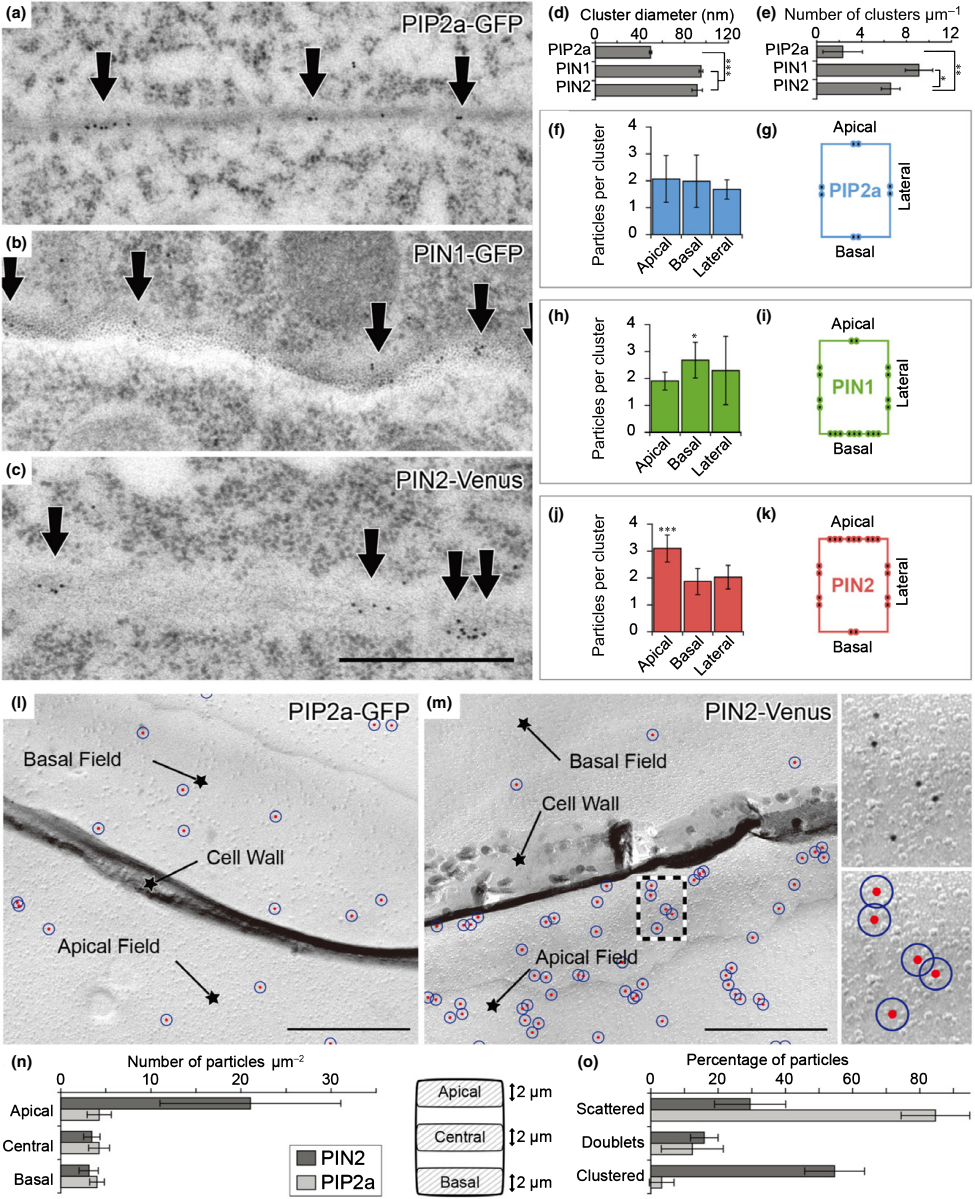
Visualization and quantification of *Arabidopsis* PIN clusters by immunogold electron microscopy (IEM) and sodium dodecyl sulfate‐digested freeze‐fracture replica labeling (SDS‐FRL). (a–c) IEM analysis with GFP antibody on high‐pressure frozen 3‐d‐old root tips in *PIP2a‐GFP* (a), *PIN1‐GFP* (b) and *PIN2‐Venus* (c). Arrows indicate the gold particles. Bar, 500 nm. (d) Quantitative analysis of the cluster diameter in *PIP2a‐GFP* (50.1 ± 1.1 nm), *PIN1‐GFP* (95.9 ± 1.7 nm) and *PIN2‐Venus* (92.4 ± 4.7 nm) based on the gold particles from IEM. Ten representative images from each line were quantified. (e) Quantification of the number of clusters with radii >55 nm in *PIP2a‐GFP* (2.31 ± 1.7), *PIN1‐GFP* (9.1 ± 1.2) and *PIN2‐Venus* (6.5 ± 0.8). Ten representative images from each line were quantified. (f–k) Quantitative analysis of IEM particles in *PIP2a‐GFP*, *n* = 18 (f, g), *PIN1‐GFP,* *n* = 15 (h, i), and *PIN2‐Venus*, *n* = 9 (j, k) at apical, basal and lateral polar domains. Average particle number per cluster (f, h, j). PIN1 and PIN2 both show significant differences in average particle number per cluster between apical and basal domains (h, j), and schematic cluster distribution (g, i, k) in single root cells are presented. (l) Specific PIP2a‐GFP immunolabeling for epidermal cells at the plasma membrane leaflet facing the protoplasm (membrane P‐face) in SDS‐FRL. The PIP2a‐GFP labeling in epidermal cells is similar in apical and in basal fields. Bars, 500 nm. (m) Characterization of PIN2‐Venus distribution in SDS‐FRL. Immunogold particles labeling GFP (10‐nm gold). Many more particles can be detected in apical fields compared to basal fields. Particles are nonhomogeneously distributed in the plasma membrane, forming loose aggregations and tight clusters. For quantitative analysis, gold particles were marked in red. A 27.5‐nm‐radius circle around the particle center was drawn in blue (enlargement of the boxed area on the right). Clusters were defined as particles residing within overlapping blue circles (center‐to‐center distance ≤ 55 nm). Bar, 500 nm. (n) Quantification of immunogold particle densities in *PIN2‐Venus* and *PIP2a‐GFP* epidermal cells in SDS‐FRL. In *PIN2‐Venus* cells the number of particles per area was significantly higher in domains close to the apical surface (distance ≤ 2 µm from the apical edge) compared to central (2 µm around the midline) and basal domains (distance ≤ 2 µm from the basal edge) (apical: 21.1 ± 10.2, central: 3.3 ± 0.8, basal: 3.1 ± 1.0). In *PIP2a‐GFP* cells numbers were similar in all domains (aplical 3.2 ± 1.8, central 2.5 ± 1.1, basal 2.7 ± 1.5). (o) Characterization of immunogold particle distribution in *PIN2‐Venus* and *PIP2a‐GFP* epidermal cells. Scattered, single particles; doublets, two particles with a center‐to‐center distance ≤55 nm; clusters, at least three particles with a nearest‐neighbor distance ≤55 nm. PIN2: scattered: 29.4 ± 10.6, doublets: 15.9 ± 6.1, clustered: 54.7 ± 12.8; PIP2a: scattered: 84.5 ± 10.2, doublets: 12.3 ± 9.2, clustered: 3.2 ± 3.7. Values are means ± SD. Mann–Whitney *U*‐test: *, *P* < 0.1; **, *P* < 0.01; ***, *P* < 0.001.

To analyze the distribution of proteins at a higher resolution, we examined *PIP2a‐GFP*, *PIN1‐GFP* and *PIN2‐Venus* by postembedding immunogold electron microscopy (IEM) (Fig. 2a–c). With this procedure, the gold particle localization does not represent directly the epitope localization, because the technique is based on indirect antigen immunodetection. Hence, gold particles may be as far as 20 nm from its epitope, due to the size of the interspersed immunoglobulins and Fab′ fragments. We defined clusters when the distance of more than two gold particles near their closest neighbor was <55 nm (Fig. S2a). This criterion is based on epitope‐to‐label distances, estimates from protein interactions and protein sizes themselves (Casuso *et al*., 2010). This quantification principle was applied to the subsequent IEM experiments. We could identify clear differences in cluster diameters and cluster density, namely both polar markers *PIN1‐GFP* and *PIN2‐Venus* had larger and denser clusters than the nonpolar marker *PIP2a‐GFP* (Fig. 2d,e).

The PIN family proteins are known to exhibit distinct membrane distributions in roots (Feraru & Friml, 2008; Luschnig & Vert, 2014). PIN1 and PIN2 represent basally and apically localized proteins, respectively. To dissect the density of polar cargoes in different polar domains at the subcellular level, we used IEM to quantify the particle numbers per cluster (Fig. 2f,h,j) as well as the overall number of immune particles (Fig. S2c–h). Clusters were assigned to apical or basal domains based on their positions relative to the cell wall (Fig. S2b). These parameters were compared at the apical (shoot apex‐facing), basal (root apex‐facing) and lateral surfaces in *PIP2a‐GFP* (Fig. 2f), *PIN1‐GFP* (Fig. 2h) and *PIN2‐Venus* (Fig. 2j). In the nonpolar *PIP2a‐GFP* control, the particles were almost evenly distributed along the membrane. In *PIN1‐GFP* and *PIN2‐Venus*, the particles and clusters were preferentially located at the basal and apical sides, respectively. At the lateral side, the PIN1 and PIN2 cluster densities decreased along a gradient from the basal and apical domains, respectively, as schematically depicted (Fig. 2g,i,k). In the case of *PIN2‐Venus*, the number of particles per cluster also varied along this gradient (Fig. 2j). The results show that the distribution of the PIN clusters is most prominent at the polar domains and gradually decreases along the lateral side away from the polar domain.

### Distribution of PIN proteins revealed by SDS‐FRL

To obtain semiquantitative data on cluster density and arrangement within the whole PM sheet, we additionally applied SDS‐FRL (Figs 2l,m, S3). This procedure facilitates the localization of integral membrane proteins beyond the limitations of thin‐section electron microscopy and reveals views of large cell membrane surfaces. For technical reasons, root samples were fractured only in the longitudinal plane (Fig. S3a) and, hence, apical and basal cell surfaces remained largely inaccessible for immunocytochemical investigations. Therefore, the density of immunoparticles (anti‐GFP) in *PIN2‐Venus* was analyzed within lateral domains of epidermal cells close to the apical surface (distance < 2 µm from the apical edge), in centrolateral regions, and close to the basal surface (distance < 2 µm from the basal edge) (Fig. 2n,o). On average, 21.1 ± 10.2 immunoparticles per µm^2^ were detected in apical domains. The overall particle density was significantly reduced in central domains with 3.3 ± 0.8 particles per µm^2^ and in domains close to the basal surface with 3.1 ± 1.0 particles per µm^2^ (area analyzed each = 100 µm^2^). In PIP2a, numbers were similar in all domains (apical 3.2 ± 1.8, central 2.5 ± 1.1, basal 2.7 ± 1.5) (Fig. 2n). Immunoparticles were nonhomogenously distributed throughout the epidermal PM, and tight clusters (particle doublets within 55 nm from each other; mean distance = 32.9 ± 10.6 nm) as well as large particle aggregations were often observed (Fig. 2m,o). Such clusters were absent in control experiments without primary antisera, indicating that the conjugated antibodies and gold particles themselves did not aggregate. The Venus epitope of the PIN2‐Venus fusion protein only appeared at the protoplasmic side (P‐face) of the membrane (Fig. S3c), which is characterized by a coarse appearance, and were absent in endodermis and stele cell types, where *PIN2* is not expressed (Fig. S3d), demonstrating the immunolabeling specificity. The immunolabeling of nonpolar PIP2a appeared at the membrane P‐face, verifying labeling specificity, and was almost evenly distributed within the different cell domains. In most cases (84.5 ± 10.2%) individual (scattered) particles were observed, while clusters (particle doublets within 55 nm from each other; mean particle distance = 31.5 ± 11.9 nm) were rarely observed (12.3 ± 9.2%). Only a few clusters (3.2 ± 3.7%) showed three to five particles (area analyzed each = 100 µm^2^) (Fig. 2o). By contrast, PIN2 showed only 29.4 ± 10.6% of scattered, 15.9 ± 6.1% of doublets and 54.7 ± 12.8 of clustered particles. Taken together, the SDS‐FRL data were consistent with the confocal microscopy observations and post‐embedding IEM quantification, further confirming the PIN protein clustering and PIN2 distribution gradient along the lateral sides.

### PIN2 clusters are independent of several cell surface structures

The localization of PIN2 in distinct clusters at the PM suggests a possible association with the cell wall, the cytoskeleton or membrane structures. Plasmodesmata that function in cell‐to‐cell communication are among the structure types confined to discrete spots within the membrane (Brunkard *et al*., 2015). No PIN2 colocalization with plasmodesmata was found by PIN2 immunolocalization in the marker line *35S::P30‐GFP* (Kim *et al*., 2005) (Fig. S4a). Colocalization of PIN2 clusters could also not be detected by aniline blue staining (Stone *et al*., 1984) of callose, a plant polysaccharide deposited at plasmodesmata and with multiple functions in growth, development and stress response (De Storme & Geelen, 2014) (Fig. S4b). Moreover, Nile red staining was used to discover intracellular lipid droplets (Greenspan *et al*., 1985) to observe whether PIN clustering was associated with their lipid ester core rather than with the membrane phospholipid. Again, no PIN2 clusters colocalized with these droplets (Fig. S4c). There was no pronounced colocalization with other proteins known to be localized in membrane microdomains such as Remorin1.2 (Fig. S4d) using immunolocalization; however, it is unclear how well the immunolocalization protocol preserves the cluster localization of nonmembrane integral proteins. The absence of PIN colocalization with these features suggests that PIN clusters may represent a unique, so far unknown, structure distinct from many of the recognized cell‐wall and membrane structures.

### PIN clustering and polar distributions are related to PIP pathways

**Fig. 3:**
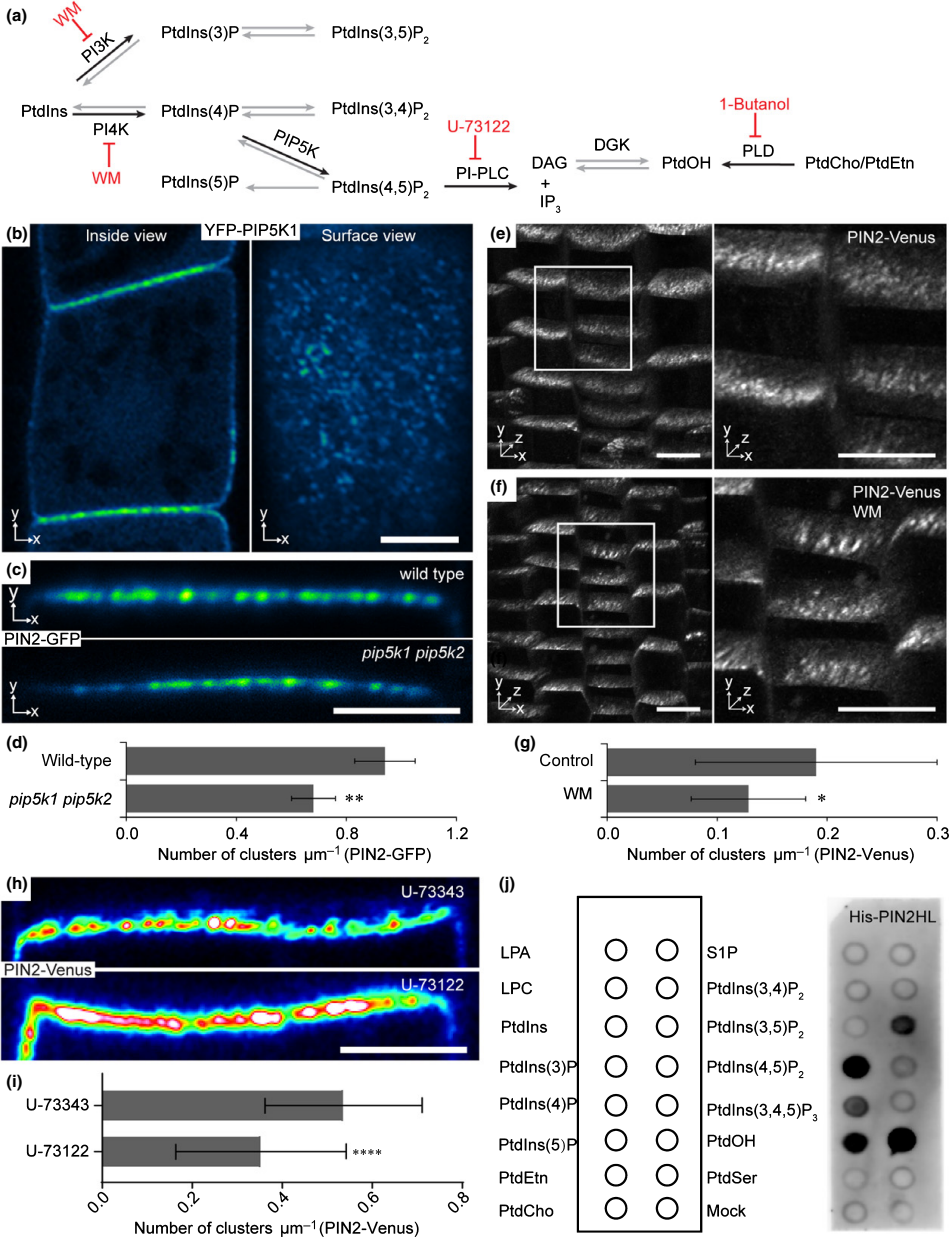
Decreased *Arabidopsis* PIN2 cluster formation by the inhibition of PIP5K, PI3K/PI4K and PI‐PLC. (a) Biosynthetic pathways of PIPs and PtdOH. Black arrows indicate the biosynthetic steps investigated in this study; the chemical inhibitors of the pathways are highlighted in red. PtdIns, phosphatidylinositol; PtdOH, phosphatidic acid; PtdCho, phosphatidylcholine; PtdEtn, phosphatidylethanolamine; PtdIns(3)P, phosphatidylinositol (3)‐phosphate; PtdIns(4)P, phosphatidylinositol (4)‐phosphate, PtdIns(5)P, phosphatidylinositol (5)‐phosphate; PtdIns(3,5)P2, phosphatidylinositol (3,5)‐bisphosphate; PtdIns(3,4)P2, phosphatidylinositol (3,4)‐bisphosphate; PtdIns(4,5)P2, phosphatidylinositol (4,5)‐bisphosphate; DAG, diacylglycerol; WM, wortmannin. (b) Clustering of *YFP‐PIP5K1* (*XVE>>YFP‐PIP5K1*) observed from inside (apical/basal domains) and surface views (lateral domain). Bar, 5 µm. (c) Clusters of PIN2 in wild type and *pip5k1 pip5k2* mutant. Bar, 2 µm. (d) Quantitative analysis of cluster density in *PIN2‐GFP* (0.95 ± 0.11) and *pip5k1 pip5k2 PIN2‐GFP* (0.68 ± 0.08). The number of clusters was manually counted and compared to the apical cell size in two dimensions to determine the cluster density. Experiments were repeated three times and in each experiment at least nine different cells from three different root tips were analyzed. Values are means ± SD. Student’s *t*‐test: **, *P* < 0.05. (e) Three‐dimensional view of PIN2‐Venus clustering as control (left) and enlargement of the boxed region (right). Bars, 10 µm. (f) Three‐dimensional view of *PIN2‐Venus* clustering after WM treatment to inhibit the PI3K and PI4K pathways. Enlargement of the boxed region (right). Bars, 10 µm. (g) Quantitative analysis of the WM treatment. The cluster density of PIN2‐Venus in control, 0.19 ± 0.11; and WM treatmentm 0.13 ± 0.05 (f). Values are means ± SD. Mann–Whitney *U*‐test: *, *P* < 0.01. (h) Clusters of PIN2 after treatment of U‐73122 to inhibit PI‐PLC activity. U‐73343, an inactive analog of U‐73122, was used as a control. Bar, 2 µm. (i) Quantitative analysis of the U‐73122 and U‐73343 treatment. The cluster density of PIN2‐Venus (U‐73343: 0.54 ± 0.17, U‐73122: 0.35 ± 0.19). Values are means ± SD. Unpaired *t*‐test: ****, *P* < 0.0001. (j) His‐PIN2HL binds to various phospholipids in a lipid binding assay using PIP strips incubated with His‐tagged PIN2 hydrophilic loop (His‐PIN2HL) and detected by anti‐His antibody. The bound protein was detected using SuperSignal western detection reagents in an Amersham 600RGB molecular imaging system (GE Healthcare) with exposure time for 20 s. LPA, lysophosphatidic acid; LPC, lysophosphocholine; S1P, sphingosine 1‐phosphate; PtdIns(3,4,5)P_3_, phosphatidylinositol (3,4,5)‐trisphosphate; PtdSer, phosphatidylserine.

The existence of specialized microdomains in the plasma membrane has been popularized by the concept of lipid or membrane rafts (Malinsky *et al*., 2013). Although phosphoinositide (PI) lipids are in low abundance, they are crucial for cell polarity and plant development (Xue *et al*., 2009; Tejos *et al*., 2014; Heilmann, 2016) as well as PIN‐dependent auxin transport (Tan *et al*., 2020b). PIs are synthesized from phosphatidylinositol (PtdIns) via phosphorylation by distinct lipid kinases and are hydrolyzed by phospholipases (Fig. 3a; Mueller‐Roeber & Pical, 2002; Meijer & Munnik, 2003; Heilmann, 2016). PtdOH is formed either directly from the hydrolysis of PtdCho/PtdEtn by phospholipase D (PLD) or from the hydrolysis of PIs by phosphoinositide phospholipase C (PI‐PLC) and subsequent phosphorylation by diacylglycerol kinase (DGK) (Fig. 3a; Rhee & Bae, 1997; Mueller‐Roeber & Pical, 2002; Meijer & Munnik, 2003; Heilmann, 2016).

To investigate whether the phospholipid composition of the membrane is involved in PIN2 clustering, we examined PIN2 clusters in lipid kinase mutants or after treatment with PIs and PtdOH biogenesis inhibitors. The phosphatidylinositol 4‐phosphate (PtdIns4P) 5‐kinases PIP5K1 and PIP5K2 have been reported to be redundantly required for the polar localization of PIN proteins. In the *pip5k1^−/−^ pip5k2^−/−^* (*pip5k1 pip5k2*) double mutant, the PIN protein distribution is partially apolar (Ischebeck *et al*., 2013; Tejos *et al*., 2014). As PIP5K1 is located at the apical and basal membrane (Ischebeck *et al*., 2013; Tejos *et al*., 2014), we noted that *YFP‐PIP5K1* formed visible aggregates at the cell surface (Fig. 3b), suggesting a close link between the PIP5K function and polarity and clustering at the cell surface. To test whether the PIP5K function in polarity is related to clustering of PIN2, we examined PIN2 clusters in *pip5k1 pip5k2* mutants (Fig. 3c). The number and density of the PIN2 clusters decreased significantly in *pip5k1 pip5k2* (Fig. 3d; Table S1), suggesting this phosphoinositide pathway contributes to PIN2‐GFP clustering.

Additionally, treatment with the lipid kinase inhibitor WM, which inhibits the activity of phosphoinositide‐3 (PI3K) and phosphoinositide‐4 (PI4K) kinases (Jaillais *et al*., 2006; Fujimoto *et al*., 2015), significantly decreased the PIN2‐Venus cluster numbers (Fig. 3e–g). Moreover, after treatment with U73122, a widely used PI‐PLC inhibitor, PIN2‐Venus clusters decreased compared to treatment with U73343, an inactive analog of U73122 (Fig. 3h,i). By contrast, inhibition of PLD‐dependent PtdOH biosynthesis with the PLD‐specific inhibitor 1‐butanol did not affect PIN2‐Venus clustering, similar to 2‐butanol, a noninhibitory analog of 1‐butanol (Fig. S4e,f). Overall, the results suggest that lipid kinase pathways are crucial for clustering of polar cargoes; in particular, PtdIns(4)P, PtdIns(4,5)P_2_, the derivatives of PtdIns(4,5)P_2_ as well as PIP5K, which is itself clustered at the PM, are required for maintaining PIN clusters.

Peripheral membrane proteins are recruited to the plasma membrane via lipid/protein or protein/protein interaction. To test whether PIN2 protein binds to the lipids directly, we performed the lipid‐binding assay of His‐tagged PIN2 hydrophilic loop (His‐PIN2HL) using PIP strips. We found that His‐PIN2HL binds to various phospholipids, including PtdIns(3)P, PtdIns(4)P, PtdIns(5)P, PtdIns(4,5)P_2_, PtdIns(3,5)P_2_ and PtdOH (Fig. 3j).

Together, these findings suggest that PIN2 protein might be recruited to some specialized microdomains by the direct interaction with phospholipids.

### Cytoskeleton requirements for clustering

**Fig. 4:**
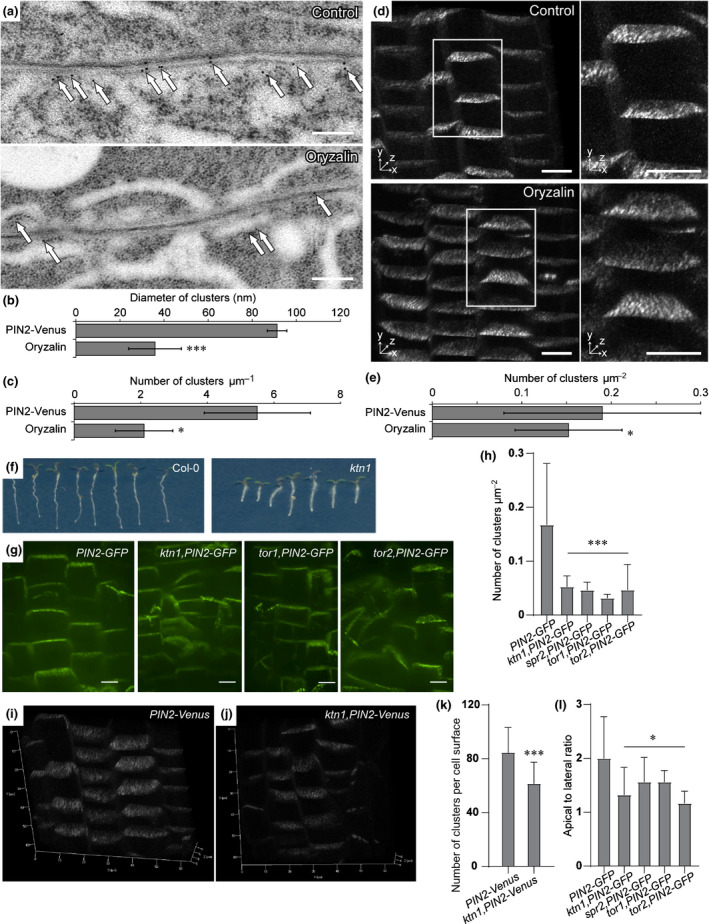
Decreased *Arabidopsis* PIN clustering by cytoskeleton breakdown. (a) Immunogold electron microscopy (anti‐GFP) in *PIN2‐Venus* after disruption of microtubules by oryzalin. The arrows point at the gold particles that represent PIN2‐GFP signals. Bar, 200 nm. (b) Quantitative analysis of the cluster diameter (control: 91.3 ± 4.4, oryzalin: 35.9 ± 11.9) and (c) cluster density (control: 5.5 ± 1.6, oryzalin: 2.1 ± 0.86) of IEM images. (d) Three‐dimensional view of confocal images of *PIN2‐Venus* clusters before and after oryzalin treatment (left), and enlargement of the boxed regions (right). Bars, 10 µm. (e) Quantitative analysis of the cluster density (control: 0.19 ± 0.11, oryzalin: 0.15 ± 0.06) in (d). Values are means ± SD. Mann–Whitney *U*‐test: *, *P* < 0.1; ***, *P* < 0.001. (f) Phenotype of 3‐d‐old seedlings of katanin mutant. (g) Confocal images of PIN2 clusters and polarity in microtubule mutants *ktn1‐5*, *tor1‐1* and *tor2‐1*. Bars, 10 µm. (h) Quantification of cluster density in microtubule mutants (PIN2‐GFP control: 0.17 ± 0.11, *n* = 95; *ktn1‐5*: 0.05 ± 0.01, *n* = 44; *spr2‐2*: 0.05 ± 0.01; *n* = 30, *tor1‐1*: 0.03 ± 0.006, *n* = 19; *tor2‐1*: 0.05 ± 0.016, *n* = 54). (k) Quantification of total size cluster numbers per cell surface in *ktn1‐5* (control: 85 ± 18.67, *n* = 30; *ktn1‐5*: 61.5 ± 15.97, *n* = 13). (l) Quantification of PIN2 polarity by apical to lateral ratio; an average of 42 cells from four seedlings were quantified for each mutant. (i) Three‐dimensional view of *PIN2‐Venus* clustering as a control. (j) Three‐dimensional view of *ktn1‐5, PIN2‐Venus* clustering.

Proteins that associate with the plasma membrane can interact with and be immobilized by association with the cell wall, the cytoskeleton or both (McKenna *et al*., 2014). The microtubule cytoskeleton determines the pattern of cellulose deposition in the cell wall, which, in turn, has been shown to affect protein diffusion within the PM (Martinière *et al*., 2012). Therefore, we examined the structural role of the cytoskeleton in PIN clustering. First, the cytoskeleton was disrupted by oryzalin treatment to depolymerize microtubules and the effect on PIN2‐Venus was observed by IEM (Fig. 4a) and confocal microscopy (Fig. 4d). Quantitative analyses revealed that in both methods the cluster size and density of clusters decreased after disruption of the microtubule network (Fig. 4b,c,e). Moreover, we observed PIN2 clusters in several microtubule mutants. Katanin is a microtubule‐severing protein with a role in microtubule organization and dynamics (Luptovčiak *et al*., 2017). SPIRAL2/TORTIFOLIA 1 (SPR2/TOR1), a plant‐specific microtubule‐associated protein, regulates the orientation of cortical microtubules and the direction of organ growth as well as the severing activity of katanin (Buschmann *et al*., 2004; Wightman *et al*., 2013). TUBULIN ALPHA‐4/TORTIFOLIA 2 (TUA4/TOR2) encodes the α‐tubulin subunit that consists of microtubule polymers (Elliott & Shaw, 2018). The *tortifolia1* (*tor1‐1*, Buschmann *et al*., 2009), *spiral2* (*spr2‐2*, Shoji *et al*., 2004) and *tortifolia2* (*tor2‐1*, Buschmann *et al*., 2009) mutants display right‐handed helical growth. The density of clusters was decreased and lateral diffusion of PIN2 was increased in the katanin mutant *ktn1‐5* (Lin *et al*., 2013), *tor1‐1*, *tor2‐1* (Buschmann *et al*., 2009) and *spr2‐2* (Shoji *et al*., 2004) (Fig. 4f–l). In addition, it has previously been shown that microtubules can control PIN2 via the CLASP‐SNX1 pathway, which controls PIN2 recycling (Ambrose *et al*., 2013). The study showed that elimination of microtubules with oryzalin could increase the amount of PIN2 in the lytic vacuoles and, hence, reduce the amount at the plasma membrane (Ambrose *et al*., 2013). This is consistent with our observation that there are fewer clusters after disrupting microtubules by oryzalin treatment. Besides changing the clustering, we also observed a stop in growth after the chemical treatments. To test whether a change in PIN clustering is not simply caused by a stop in growth, we treated roots with AlCl_3_, a known inhibitor of growth (Yang *et al*., 2014). We did not observe a change in clustering after 1, 2 or 3 h of AlCl_3_ treatment (Fig. S5a). Disruption of the actin network with Latrunculin B treatment to inhibit actin polymerization did not reduce the clustering significantly (Fig. S5b,c). Thus, we conclude that microtubules are involved in the clustering of PIN2.

### Cell‐wall components required for clustering

**Fig. 5:**
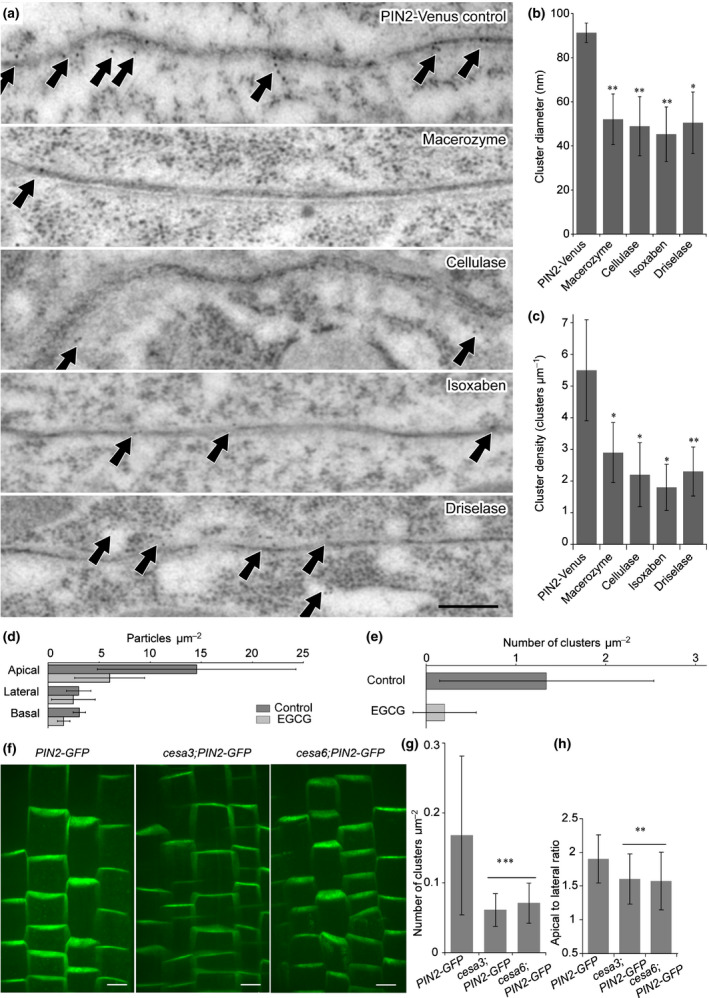
Decreased *Arabidopsis* PIN2 clustering by cell‐wall component disruption. (a) Immunogold electron microscopy of decreased *PIN2‐Venus* clustering after isoxaben treatment or digestion of specific cell‐wall components with macerozyme, cellulase and driselase. White arrows indicate the PIN2 clusters. Bar, 200 nm. (b, c) Quantitative analysis of the cluster diameter (b) and density (c) after cell wall disruption. Values are means ± SD. Mann–Whitney *U*‐test: *, *P* < 0.1; **, *P* < 0.01. (d, e) SDS‐FRL quantification of immunogold particle densities in *PIN2‐Venus* epidermal after 24 h of treatment with 50 µM EGCG. (d) The number of particles µm^–2^ is lower after EGCG treatment in domains close to the apical surface (EGCG: apical: 14.6 ± 9.6, central: 3.2 ± 1.4, basal: 3.1 ± 1.3; control: apical: 6.0 ± 3.5, central: 1.6 ± 0.6, basal: 2.6 ± 2.2). (e) Number of clusters µm^–2^ is lower after EGCG treatment in apical domains (EGCG: 0.20 ± 0.35; control: 1.34 ± 1.20). (f) Confocal images of PIN2 clusters and polarity in cellulose mutants. Bars, 10 µm. (g) Quantification of cluster density in the cellulose mutants. Number of clusters µm^−2^ are lower in *cesa3* and *cesa6* mutants than in control (control: 0.17 ± 0.11, *cesa3*: 0.06 ± 0.02, *cesa6*: 0.07 ± 0.02). Values are means ± SD. Mann–Whitney *U*‐test: ***, *P* < 0.001. (h) Quantification of PIN2 polarity by apical to lateral ratio in the mutants; an average of 40 cells from four seedlings were quantified for each mutant. Values are means ± SD. Mann–Whitney *U*‐test: **, *P* < 0.01.

The cell wall and its constituents, such as cellulose produced by the cellulose synthase catalytic subunit (CESA), are needed for PIN polarity maintenance (Feraru *et al*., 2011; Martinière *et al*., 2012). To test whether cell‐wall components might play a role in regulating PIN clusters, we utilized pharmacological treatments known to disrupt specific components of the cell wall, such as macerozyme (pectin depolymerase), cellulase (cellulolysis catalysis), isoxaben (synthesis inhibitor of cell‐wall materials) and driselase (fungal enzyme mixture, including cellulase, pectinase, xylanase and mannanase). After application, *PIN2‐Venus* clusters were decreased in IEM labeling (Fig. 5a). As a control, we performed the same chemical treatments using PIP2a‐GFP‐expressing plants to see whether the chemicals influence health of the cells or GFP‐fluorescence and found a normal appearance of plasma membrane signal after all treatments (Fig. S6). Quantitative analysis indicated that disruption of cell‐wall components led to significant decreases in both PIN cluster size and density (Fig. 5b,c). To confirm this observation, we used the SDS‐FRL method to look at PIN2‐Venus and wild type plants treated with EGCG, a specific inhibitor of a pectin methyl esterase (Wolf *et al*., 2012b). After 24 h of treatment with 50 µM EGCG, we measured a significantly lower particle density in the apical domain (EGCG: 6.0 ± 3.5, control: 14.6 ± 9.6 clusters µm^−2^) (Fig. 5d), as well as fewer clusters (EGCG: 0.20 ± 0.35 cluster µm^−2^) compared to the control (1.34 ± 1.20 cluster µm^−2^) (Fig. 5e). As suggested by the cellulase‐induced decrease in the density of PIN2 clusters, we quantified PIN2 clusters and the PIN2 apical‐to‐lateral ratio in two cellulose mutants, *cesa3* and *cesa6* (Feraru *et al*., 2011) (Fig. 5f–h). We found decreased PIN2 cluster density and coinciding PIN2 polarity defects in the cellulose mutants. Moreover, plasmolysis on *PIN2‐Venus* with 0.5 M mannitol to disrupt connections between cell walls and PM followed by IEM also slightly decreased the PIN2 cluster sizes and densities (Fig. S7), probably due to the PIN protein redistribution and PIN2 cluster accumulation on hechtian strands, a stretched PM extending from the plasmolyzed protoplast to the cell wall (Fig. S7a). Together, we found that general cell‐wall components, such as cellulose, pectin, as well as the connection between PM and cell wall, are important for PIN clustering.

### The integrin‐like protein NDR1 is involved in PIN2 clustering and polarity

**Fig. 6:**
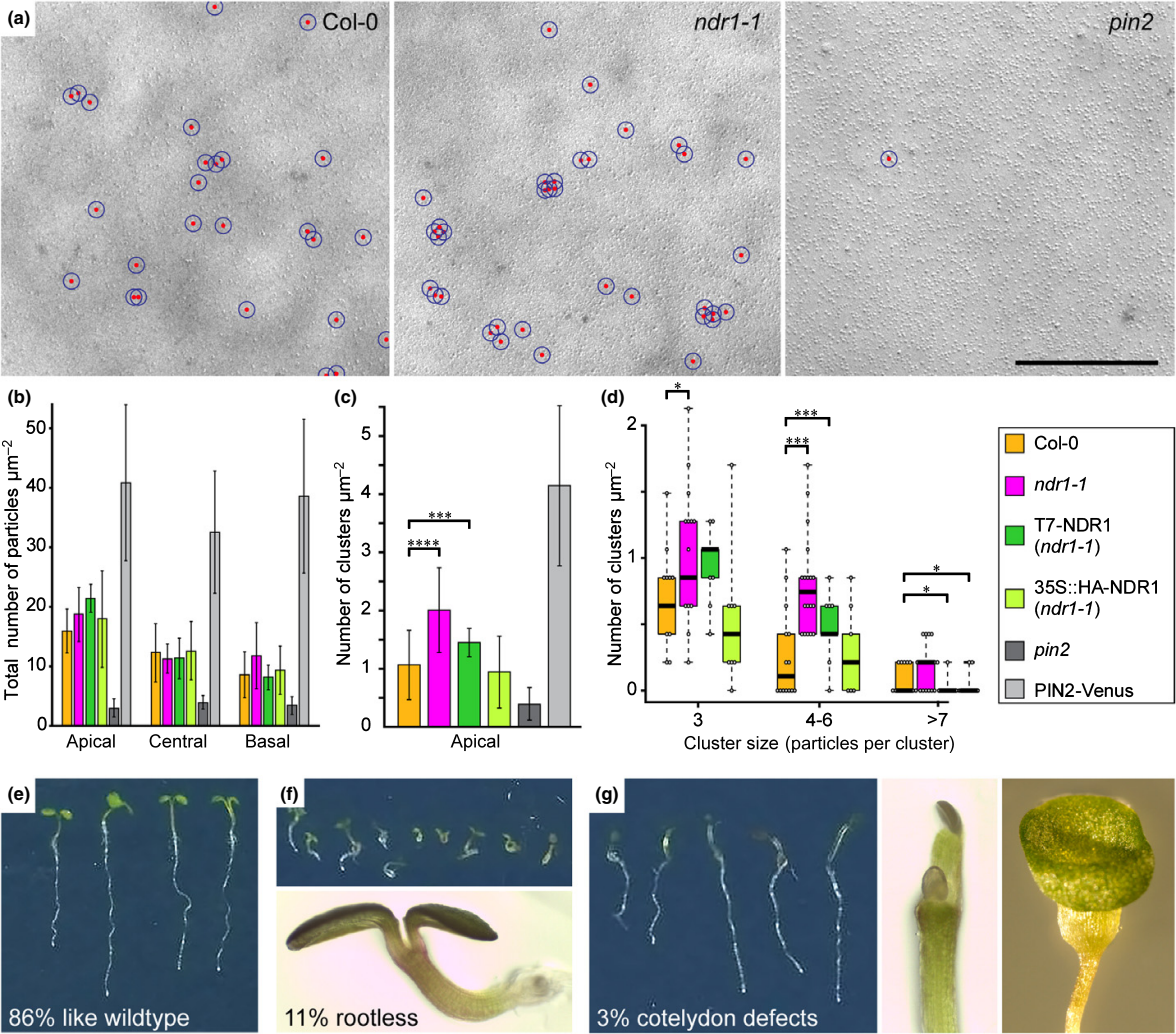
PIN2 clustering affected by the *Arabidopsis ndr1* mutant defective in plasma membrane–cell wall adhesion. Quantitative analysis of the anti‐PIN2 immunoparticle density in SDS‐FRL images of *ndr1‐1* mutant and the complementation lines T7‐NDR1 (native promotor) and 35S::HA‐NDR1 (overexpression), and controls: wild type (Col‐0), *pin2* mutant and PIN2‐Venus. (a) Example SDS‐FRL images of wild type (Col‐0), *ndr1‐1* mutant and *pin2* mutant. For each line at least 10 cells in five plants were analyzed. Bar, 500 nm. (b) Quantitative analysis of the total number of particles in different intracellular domains (apical, central, basal). (c) Cluster density (cluster = more than three particles ≤55 nm from each other) in the apical domain. The *ndr1‐1* line shows almost double the number of clusters per area compared to the wild type. (d) Cluster size in the apical domain. (e) Normal phenotype of 6‐d‐old *ndr1‐1* mutant seedling and (f) rootless phenotype of *ndr1‐1*, overview (upper image) and enlarged view of a rootless seedling (lower image). (g) Cotyledon defects phenotype of *ndr1‐1* (left) and enlarged views (right). Values are means ± SD. Mann–Whitney *U*‐test: *, *P* < 0.1; ***, *P* < 0.001; ****, *P* < 0.0001.

The *Arabidopsis* NON‐RACE‐SPECIFIC DISEASE RESISTANCE1 (NDR1) is a PM‐localized protein with conserved motifs, suggesting homology with mammalian integrins that are well‐characterized proteins involved in adhesion and signaling (Knepper *et al*., 2011). The *ndr1‐1* mutant is defective in adhesion between the cell wall and PM (Knepper *et al*., 2011). To test whether this protein is involved in PIN2 clustering we performed a SDS‐FRL experiment using an anti‐PIN2 antibody to measure the cluster density in the *ndr1‐1* mutant compared to wild type. First, the overall particle density was similar to the wild type and showed the typical enrichment in the apical fields (Fig. 6a,b). We counted the number of clusters in the apical field and found that there were significantly more clusters in the *ndr1‐1* mutant compared to the wild type (Fig. 6c). Furthermore, the *ndr1‐1* mutant appeared to have clusters composed of more particles compared with the wild type (Fig. 6d). In this experiment, we included two complementation lines, one line expressing NDR1 under its native promotor and an overexpression line using the 35S promotor. The native promotor line was able to rescue the phenotype only to a certain extent, while the overexpression line fully rescued the number of clusters as well as the size of clusters (Fig. 6c,d). The morphological phenotypes of the *ndr1‐1* mutant showed an increased frequency of abnormal roots (11% rootless) and cotyledon development (3% cotyledon defects) (Fig. 6e–g). These phenotypes were similar to those found in multiple *pin* mutants or mutants defective in PIN polarity (Friml *et al*., 2003, Friml *et al*., 2004). These results indicate that NDR1 and the connection between PM and the cell wall play important roles in PIN clustering and polarity maintenance.

### Conclusions

Outside the plant kingdom, various mechanisms and processes have been shown to play a role in cell polarity maintenance and polar cargo distribution within PM polar domains, among which the tight junction‐based diffusion barriers bordering the polar domains are prominent (Mellman & Nelson, 2008). Most plant cells have no similar diffusion barriers, but, unlike their animal counterparts, are surrounded by an extracellular matrix – the cell wall. Previously, we demonstrated that plant polar cargoes, exemplified by polarly localized PIN auxin transporters, are distributed at the polar domains where they are grouped into largely immobile clusters. Computational modeling suggests membrane super‐polar exocytosis and lateral endocytosis may contribute to PIN polar localization and lateral diffusion (Kleine‐Vehn *et al*., 2011). However, the cellular processes underlying the formation of PIN clusters as well as the role of these clusters in polarity maintenance are unknown. Here, we reveal multiple insights into PIN protein clustering – we visualized it in different cells and at different polar domains, identified several necessary cellular structural features, and provided strong, correlative support for the importance of clusters in cell polarity.

### Visualization and quantification of clusters in *Arabidopsis*

Real‐time visualization of cluster formation and maintenance in plant cells is challenging due to the high *Z*‐resolution required to monitor the apical and basal cell surfaces lying perpendicular to the focal plane. However, the quantitative analysis of clusters is necessary to obtain insights into when and how the clusters are formed and what their lifetime is. We report on the quantification of polar cargo clustering by confocal microscopy and high‐resolution electron microscopy methods, specifically post‐embedding immunogold and SDS‐FRL. The latter approach allows the immunolabeling of integral membrane proteins without diffusion restrictions and their quantification within large fields of the PM in a pseudo‐3D manner. These approaches complemented each other to show the following: a clustering of the PIN1 and PIN2 polar cargoes, which contrasts with the more uniformly distributed nonpolar cargoes; a stronger bias towards clustering in the polar domains; and an inverse correlation between the clustering gradient and the distance from the polar domain.

The number of particles per cluster may affect the generation of polar distribution gradients. For instance, in fission yeast, the dual‐specificity tyrosine phosphorylation‐regulated kinase (DYRK)‐type protein kinase pom1p associates with the PM at the cell tip and diffuses laterally on the membrane, while aggregating in clusters and dissociating from the membrane after cluster fragmentation (Saunders *et al*., 2012). We also identified a correlation between the number of PIN particles per cluster and the concentration of PIN along its polar gradient. It remains unclear whether this correlation implies functional roles for variations in PIN cluster size, such as altered lateral diffusion or stabilization within the PM.

### PIs are important for PIN clustering

The PtdIns4P 5‐kinases, PIP5K1 and PIP5K2, have been reported to be redundantly required for the polar localization of PIN proteins (Ischebeck *et al*., 2013; Tejos *et al*., 2014) and the polar localization of PIP5K to possibly facilitate the localized synthesis of PtdIns(4,5)P_2_ in both plants and animals. Notably, we observed that PIP5K1 forms visible aggregates at the cell surface similar to PIN clusters. Furthermore, PIN2 cluster density decreased significantly in the *pip5k1 pip5k2* mutant, suggesting a close link between PIP5K lipid kinase function and polarity and polar cargo clustering. Additionally, pharmacological interference with PI‐PLC function and with PI3K and PI4K signaling inhibited PIN2 clustering. Additional pathways known to regulate PIN polarity, such as the reversible phosphorylation of conserved PIN motifs (Huang *et al*., 2010; Zhang *et al*., 2010; Barbosa *et al*., 2014; Rakusová *et al*., 2016), *MAB4/ENP/NPY1‐LIKE* (Furutani *et al*., 2011), may similarly regulate PIN clustering, but this hypothesis remains to be tested. Overall, the results suggest that lipid kinase pathways are important for cluster maintenance; in particular, PtdIns(4)P, PtdIns(4,5)P_2_, the derivatives of PtdIns(4,5)P_2_ as well as the corresponding enzyme PIP5K, which itself shows a clustered distribution, are required for both PIN clustering and polarity, further correlating these two processes.

Interestingly, recent studies have shown that PtdIns(4)P is required for the Remorin nanodomain organization (Gronnier *et al*., 2017; Legrand *et al*., 2019; Huang *et al*., 2019) and the association of D6PK to the PM depends on the phospholipid composition and PIP5K function (Barbosa *et al*., 2016), suggesting that PIs not only affect clustering of PINs but also other important PM‐resident proteins.

Our data have also demonstrated a direct interaction of PIN2 proteins with phospholipids, including PtdIns(3)P, PtdIns(4)P, PtdIns(4,5)P_2_ and the derivatives of PtdIns(4,5)P_2_, suggesting that phospholipids might regulate PIN2 clustering by their direct interaction with PIN2 hydrophilic loops. However, visalization of PtdIns(3)P, PtdIns(4)P and PtdIns(4,5)P_2_ with their respective biosensors demonstrates that PtdIns(4)P and PtdIns(4,5)P_2_ localize to the PM and intracellular compartments while PtdIns(3)P localizes to late endosomes (Simon *et al*., 2014), indicating that PtdIns(4)P, PtdIns(4,5)P_2_ or the derivatives of PtdIns(4,5)P_2_, rather than PtdIns(3)P, play a role in PIN clustering.

In addition to PIs, the PM lipid composition such as phosphatidylserine (PS) ratios (Skotland & Sandvig, 2019), sphingolipid chain length (Markham *et al*., 2011), glycosyl inositol phosphorylceramide (GIPC) content (Gronnier *et al*., 2016) and sterol content (Tapken & Murphy, 2015) probably play an important role in nanodomain formation and funtion. Nonetheless, the exact involvement of different phospholipid membrane constituents and their possible clustered distribution within the PM are topics for further investigation.

### Cell wall and connections to the PM are required for PIN clustering

Colocalization studies and pharmacological and/or genetic interference with cellular structural components have suggested that PtdInsP‐related constituents of the membranes, microtubule cytoskeleton and cell wall, as well as connections between cell wall and PM are required for PIN clustering. Plant developmental processes, such as embryo development, anisotropic growth, directional cell elongation as well as shoot and root growth are impaired in mutants deficient in lipid kinases, microtubules, cell wall or in *ndr1‐1* (Fig. 6e–g; Shoji *et al*., 2004; Buschmann *et al*., 2009; Persson *et al*., 2007; Feraru *et al*., 2011; Tejos *et al*., 2014; Luptovčiak *et al*., 2017; Hu *et al*., 2018). Coincidently, PIN2 clustering andpolar auxin transport (Fig. S8) are also altered in lipid kinase, microtubule and *ndr1‐1* mutants. Whereas the *ndr1‐1* mutant shows increased PIN2 clustering concomitantly with increased auxin transport (Figs 6c, S8), the *pip5k1 pip5k2* and *spr2‐2* mutants show decreased PIN2 clustering but increased auxin transport (Figs 3d, 4h). Therefore, the casual connection between auxin transport and PIN2 clustering remains unclear and awaits further study. Structures surrounding the PM may act to restrict the diffusion of scaffold proteins and molecular fences, and perhaps also, through anchoring, promote their clustering within the PM (Mellman & Nelson, 2008). A variety of multicomponent linkages between the cell wall, PM and cytoskeleton are known in plants (Liu *et al*., 2015). Some fasciclin‐like arabinogalactan (FLA) proteins are thought to function in nanodomain–cell‐wall interactions (Tapken & Murphy, 2015). A membrane skeleton fence model was proposed to illustrate the regulation of PM organization (Kusumi & Sako, 1996). In the model, membrane skeleton/cytoskeleton structures control the mobility and assembly of membrane proteins in specialized domains. We speculate that this model might also describe the plant PM because several nanodomain proteins were reported to align with or bind to microtubules or actin in plants (Jarsch *et al*., 2014; Gui *et al*., 2015; Lv *et al*., 2017; Daněk *et al*., 2020). In addition, cytoskeleton disruption leads to an increased diffusion rate or reduction of nanodomains (Lv *et al*., 2017; McKenna *et al*., 2019). Through genetic and pharmacological interference, we confirmed the involvement of microtubules in PIN clustering (Fig. 4). At the same time, increased lateral diffusion of PIN2 proteins in the microtubule mutants was also detected (Fig. 4l), suggesting that a membrane cytoskeleton fence confines the movement and assembly of PIN2 clusters.

The cell wall confines the lateral diffusion of PM proteins (Martinière *et al*., 2012). Cell‐wall disruption by protoplasting increases the lateral diffusion of PM proteins, including PIN2 and PIN3 (Feraru *et al*., 2011; Martinière *et al*., 2012; McKenna *et al*., 2019). Consequently, PIN2‐GFP polarity measured by the apical to lateral signal ratio decreased in cellulose synthase mutants *cesa3* and *cesa6* (Fig. 5f,h).

NDR1 is an integrin‐like protein, which is thought to mediate the PM–cell wall adhesions/connection (Knepper *et al*., 2011; Liu *et al*., 2015). NDR1 recognizes the extracellular matrix through its NGD motif, which is similar to the RGD motif (Knepper *et al*., 2011). Cell wall–PM adhesion is altered in the *ndr1‐1* mutant (Knepper *et al*., 2011). Several studies have shown that increasing the distance between the cell wall and PM by plasmolysis promoted the lateral diffusion of PM proteins, such as PIN2 (Feraru *et al*., 2011; Martinière *et al*., 2012). Our data showed that PIN2 clustering increased in the *ndr1‐1* mutant (Fig. 6c,d). These findings demonstrated that the PM–cell wall adhesion/connection confines the mobility and assembly of PM proteins. In epithelial cells, RGD‐containing ligands bound to the integrin receptor activate the RhoA‐formin signaling pathway to generate PM nanodomains (Kalappurakkal *et al*., 2019). Thus, we speculate that there might exist a more complicated mechanism underlying PM–cell wall adhesion in the regulation of PIN clustering.

**Fig. 7:**
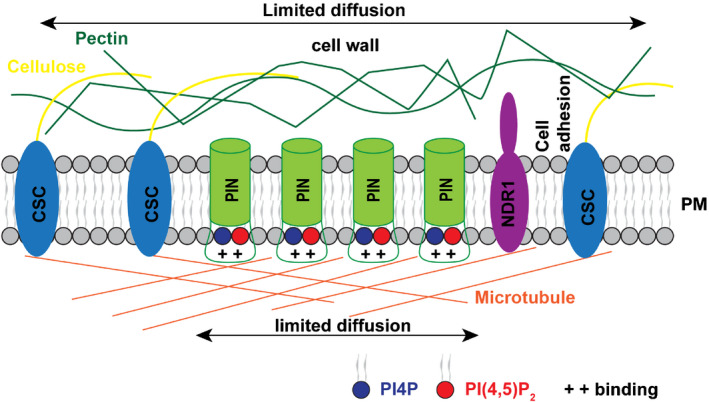
Diagram of cellular structures regulating *Arabidopsis* PIN clustering. The transmembrane PIN proteins form clusters within the plasma membrane (PM). PIN clustering is regulated by multiple structures, including PIs, microtubules, cell wall and the cell wall–PM connection through NDR1. PIs might regulate the PIN2 clustering by their direct interaction with the PIN2 hydrophilic loop. Microtubules and cell wall (cellulose, pectin) confine the lateral diffusion of PIN2 proteins. NDR1‐mediated PM–cell wall adhesion is involved in PIN clustering via an as yet unclear mechanism.

Certainly, the diversity of elements necessary for PIN clustering that has been identified here implies a complicated network of interactions to control this process (Fig. 7). However, interference with specific components of these complex structures may have resulted in their generalized impairment that might affect a specific linkage between the cell wall, PM and cytoskeleton.

Together, the use of diverse imaging methods combined with genetic and pharmacological approaches, interfering with diverse cellular processes, has provided insights into the distribution of polar cargoes (illustrated by PIN proteins) in plants. Our results indicate that the clusters of polar cargos are unique structures presumably linked to overall polarity regulation. The strong dependence of clustering on the plant cell wall and its connection to the PM suggests that plants might have evolved mechanisms of polarity maintenance distinctive from those of metazoans.

## Author contributions

HL and JF conceived the study and designed experiments. HL, DW, XZ, ST, SN, RDR, WAK, DG, MK, RT, PG, XC and JD performed the experiments. HL, ND‐M, XZ, ST, KW, DW, WAK and RT analyzed the data. ND‐M designed the quantification software for confocal images and assisted with data analysis and generation of contour and mesh plots. HL and RDR conducted the post‐embedding immunogold transmission electron microscopy experiment. HL, DW, WAK, PG and DG performed the SDS‐FRL experiments. HL, DW, KW and WAK did the TEM analysis. RT generated and analyzed the PIP5K‐related data. JD and DW carried out the colocalization experiment. ST performed the lipid binding assay. XZ carried out the auxin transport experiment. HL performed the remainder of the experiments. SN, XC and JD helped with design and discussion of the data. HL, DW, XZ and JF wrote the manuscript.

## Supporting information


**Fig. S1** Visualization and quantification of PIN clusters by confocal microscopy in different zones of the root tip.
**Fig. S2** Quantification of the polar cargo distribution in different polar domains from IEM images.
**Fig. S3** Specific PIN2 localization to epidermal and cortex cells confirmed by SDS‐FRL.
**Fig. S4** Independent PIN cluster positions from several PM structures.
**Fig. S5** Clustering after treatment with AlCl_3_ or latrunculin.
**Fig. S6** No effect of cell‐wall‐digesting chemical on PIP2a‐GFP compared to PIN2‐Venus.
**Fig. S7** *PIN2‐Venus* clustering after plasmolysis by IEM.
**Fig. S8** Increased auxin transport in hypocotyls of *spr2‐2*, *pip5k1 pip5k2* and *ndr1* mutants.
**Methods S1** SDS‐digested freeze‐fracture replica labeling.
**Table S1** Manual quantification of PIN2 clusters in the *pip5k1 pip5k2* mutant (Figure 3).

**Video S1** Three‐dimensional rotation of *PIN2‐GFP* clusters.

**Video S2** *PIN2‐GFP* root cap and epidermal cell clusters during root growth.
Please note: Wiley Blackwell are not responsible for the content or functionality of any Supporting Information supplied by the authors. Any queries (other than missing material) should be directed to the *New Phytologist* Central Office.

## References

[nph16887-bib-0001] Abas L , Benjamins R , Malenica N , Paciorek T , Wiśniewska J , Moulinier‐Anzola JC , Sieberer T , Friml J , Luschnig C . 2006. Intracellular trafficking and proteolysis of the *Arabidopsis* auxin‐efflux facilitator PIN2 are involved in root gravitropism. Nature Cell Biology 8: 249–256. 16489343 10.1038/ncb1369

[nph16887-bib-0002] Adamowski M , Friml J . 2015. PIN‐dependent auxin transport: action, regulation, and evolution. The Plant Cell 27: 20–32. 25604445 10.1105/tpc.114.134874 PMC4330589

[nph16887-bib-0003] Ambrose C , Ruan Y , Gardiner J , Tamblyn LM , Catching A , Kirik V , Marc J , Overall R , Wasteneys GO . 2013. CLASP interacts with sorting nexin 1 to link microtubules and auxin transport via PIN2 recycling in *Arabidopsis thaliana* . Developmental Cell 24: 649–659. 23477787 10.1016/j.devcel.2013.02.007

[nph16887-bib-0004] Barbosa ICR , Shikata H , Zourelidou M , Heilmann M , Heilmann I , Schwechheimer C . 2016. Phospholipid composition and a polybasic motif determine D6 PROTEIN KINASE polar association with the plasma membrane and tropic responses. Development 143: 4687–4700. 27836964 10.1242/dev.137117

[nph16887-bib-0005] Barbosa ICR , Zourelidou M , Willige BC , Weller B , Schwechheimer C . 2014. D6 PROTEIN KINASE activates auxin transport‐dependent growth and PIN‐FORMED phosphorylation at the plasma membrane. Developmental Cell 29: 674–685. 24930721 10.1016/j.devcel.2014.05.006

[nph16887-bib-0006] Baster P , Robert S , Kleine‐Vehn J , Vanneste S , Kania U , Grunewald W , De Rybel B , Beeckman T , Friml J . 2012. SCFTIR1/AFB‐auxin signalling regulates PIN vacuolar trafficking and auxin fluxes during root gravitropism. EMBO Journal 32: 260–274. 10.1038/emboj.2012.310 PMC3553380 23211744

[nph16887-bib-0007] Benková E , Michniewicz M , Sauer M , Teichmann T , Seifertová D , Jürgens G , Friml J . 2003. Local, efflux‐dependent auxin gradients as a common module for plant organ formation. Cell 115: 591–602. 14651850 10.1016/s0092-8674(03)00924-3

[nph16887-bib-0008] Brunkard JO , Runkel AM , Zambryski PC . 2015. The cytosol must flow: intercellular transport through plasmodesmata. Current Opinion in Cell Biology 35: 13–20. 25847870 10.1016/j.ceb.2015.03.003

[nph16887-bib-0009] Buschmann H , Fabri CO , Hauptmann M , Hutzler P , Laux T , Lloyd CW , Schäffner AR . 2004. Helical growth of the *Arabidopsis* mutant *tortifolia1* reveals a plant‐specific microtubuleassociated protein. Current Biology 14: 1515–1521. 15324671 10.1016/j.cub.2004.08.033

[nph16887-bib-0010] Buschmann H , Hauptmann M , Niessing D , Lloyd CW , Schäffner AR . 2009. Helical growth of the *Arabidopsis* mutant *tortifolia2* does not depend on cell division patterns but involves handed twisting of isolated cells. The Plant Cell 21: 2090–2106. 19638477 10.1105/tpc.108.061242 PMC2729594

[nph16887-bib-0011] Casuso I , Sens P , Rico F , Scheuring S . 2010. Experimental evidence for membrane‐mediated protein–protein interaction. Biophysical Journal 99: L47–L49. 20923630 10.1016/j.bpj.2010.07.028 PMC3042552

[nph16887-bib-0012] Chevalier A , Chaumont F . 2014. Trafficking of plant plasma membrane aquaporins: multiple regulation levels and complex sorting signals. Plant and Cell Physiology 56: 819–829. 25520405 10.1093/pcp/pcu203 PMC7107115

[nph16887-bib-0101] Chu YJ , Chen X , Xue HW . 2016. Ins(1,4,5)P_3_ suppresses protein degradation in plant vacuoles by regulating SNX‐mediated protein sorting. Molecular Plant 9: 1440–1443. 27477682 10.1016/j.molp.2016.07.009

[nph16887-bib-0013] Coppinger P , Repetti PP , Day B , Dahlbeck D , Mehlert A , Staskawicz BJ . 2004. Overexpression of the plasma membrane‐localized NDR1 protein results in enhanced bacterial disease resistance in *Arabidopsis thaliana* . The Plant Journal 40: 225–237. 15447649 10.1111/j.1365-313X.2004.02203.x

[nph16887-bib-0014] Craddock C , Yang Z . 2012. Endocytic signaling in leaves and roots: same rules different players. Frontiers in Plant Science 3: 219. 23060890 10.3389/fpls.2012.00219 PMC3462323

[nph16887-bib-0015] Cutler SR , Ehrhardt DW , Griffitts JS , Somerville CR . 2000. Random GFP:cDNA fusions enable visualization of subcellular structures in cells of Arabidopsis at a high frequency. Proceedings of the National Academy of Sciences, USA 97: 3718–3723. 10.1073/pnas.97.7.3718 PMC16306 10737809

[nph16887-bib-0016] Daněk M , Angelini J , Malínská K , Andrejch J , Amlerová Z , Kocourková D , Brouzdová J , Valentová O , Martinec J , Petrášek J . 2020. Cell wall contributes to the stability of plasma membrane nanodomain organization of *Arabidopsis thaliana* FLOTILLIN2 and HYPERSENSITIVE INDUCED REACTION1 proteins. The Plant Journal 101: 619–636. 31610051 10.1111/tpj.14566

[nph16887-bib-0017] Day B , Dahlbeck D , Staskawicz BJ . 2006. NDR1 interaction with RIN4 mediates the differential activation of multiple disease resistance pathways in *Arabidopsis* . The Plant Cell 18: 2782–2791. 17012600 10.1105/tpc.106.044693 PMC1626609

[nph16887-bib-0018] De Storme N , Geelen D . 2014. Callose homeostasis at plasmodesmata: molecular regulators and developmental relevance. Frontiers in Plant Science 5: 138. 24795733 10.3389/fpls.2014.00138 PMC4001042

[nph16887-bib-0019] Desprez T , Juraniec M , Crowell EF , Jouy H , Pochylova Z , Parcy F , Hofte H , Gonneau M , Vernhettes S . 2007. Organization of cellulose synthase complexes involved in primary cell wall synthesis in *Arabidopsis thaliana* . Proceedings of the National Academy of Sciences, USA 104: 15572–15577. 10.1073/pnas.0706569104 PMC2000492 17878303

[nph16887-bib-0020] Dodgson J , Chessel A , Yamamoto M , Vaggi F , Cox S , Rosten E , Albrecht D , Geymonat M , Csikasz‐Nagy A , Sato M *et al*. 2013. Spatial segregation of polarity factors into distinct cortical clusters is required for cell polarity control. Nature Communications 4: 1834. 10.1038/ncomms2813 PMC3674234 23673619

[nph16887-bib-0103] Elliott A , Shaw SL . 2018. Update: plant cortical microtubule arrays. Plant Physiology 176: 94–105. 29184029 10.1104/pp.17.01329 PMC5761819

[nph16887-bib-0021] Fagard M , Desnos T , Desprez T , Goubet F , Refregier G , Mouille G , McCann M , Rayon C , Vernhettes S , Hofte H . 2000. PROCUSTE1 encodes a cellulose synthase required for normal cell elongation specifically in roots and dark‐grown hypocotyls of *Arabidopsis* . The Plant Cell 12: 2409–2423. 11148287 10.1105/tpc.12.12.2409 PMC102227

[nph16887-bib-0022] Feraru E , Feraru MI , Kleine‐Vehn J , Martinière A , Mouille G , Vanneste S , Vernhettes S , Runions J , Friml J . 2011. PIN polarity maintenance by the cell wall in *Arabidopsis* . Current Biology 21: 338–343. 21315597 10.1016/j.cub.2011.01.036

[nph16887-bib-0023] Feraru E , Friml J . 2008. PIN polar targeting. Plant Physiology 147: 1553–1559. 18678746 10.1104/pp.108.121756 PMC2492634

[nph16887-bib-0024] Friml J , Vieten A , Sauer M , Weijers D , Schwarz H , Hamann T , Offringa R , Jürgens G . 2003. Efflux‐dependent auxin gradients establish the apical–basal axis of Arabidopsis. Nature 426: 147–153. 14614497 10.1038/nature02085

[nph16887-bib-0025] Friml J , Yang X , Michniewicz M , Weijers D , Quint A , Tietz O , Benjamins R , Ouwerkerk PBF , Ljung K , Sandberg G *et al*. 2004. A PINOID‐dependent binary switch in apical–basal PIN polar targeting directs auxin efflux. Science 306: 862–865. 15514156 10.1126/science.1100618

[nph16887-bib-0026] Fujimoto M , Suda Y , Vernhettes S , Nakano A , Ueda T . 2015. Phosphatidylinositol 3‐kinase and 4‐kinase have distinct roles in intracellular trafficking of cellulose synthase complexes in *Arabidopsis thaliana* . Plant & Cell Physiology 56: 287–298. 25516570 10.1093/pcp/pcu195

[nph16887-bib-0027] Furutani M , Sakamoto N , Yoshida S , Kajiwara T , Robert H , Friml J , Tasaka M . 2011. Polar‐localized NPH3‐like proteins regulate polarity and endocytosis of PIN‐FORMED auxin efflux carriers. Development 138: 2069–2078. 21490067 10.1242/dev.057745

[nph16887-bib-0028] Geldner N . 2009. Cell polarity in plants – a PARspective on PINs. Current Opinion in Plant Biology 12: 42–48. 18993110 10.1016/j.pbi.2008.09.009

[nph16887-bib-0029] Glanc M , Fendrych M , Friml J . 2018. Mechanistic framework for cell‐intrinsic re‐establishment of PIN2 polarity after cell division. Nature Plants 4: 1082–1088. 30518833 10.1038/s41477-018-0318-3 PMC6394824

[nph16887-bib-0030] Glanc M , Fendrych M , Friml J . 2019. PIN2 polarity establishment in *Arabidopsis* in the absence of an intact cytoskeleton. Biomolecules 9: 222. 10.3390/biom9060222 PMC6628292 31181636

[nph16887-bib-0031] Greenspan P , Mayer EP , Fowler SD . 1985. Nile red: a selective fluorescent stain for intracellular lipid droplets. Journal of Cell Biology 100: 965–973. 10.1083/jcb.100.3.965 PMC2113505 3972906

[nph16887-bib-0032] Grones P , Friml J . 2015. Auxin transporters and binding proteins at a glance. Journal of Cell Science 128: 1–7. 25556248 10.1242/jcs.159418

[nph16887-bib-0033] Gronnier J , Crowet JM , Habenstein B , Nasir MN , Bayle V , Hosy E , Platre MP , Gouguet P , Raffaele S , Martinez D *et al*. 2017. Structural basis for plant plasma membrane protein dynamics and organization into functional nanodomains. eLife 6: e26404. 28758890 10.7554/eLife.26404 PMC5536944

[nph16887-bib-0034] Gronnier J , Germain V , Gouguet P , Cacas JL , Mongrand S . 2016. GIPC: Glycosyl inositol phospho ceramides, the major sphingolipids on earth. Plant Signaling and Behavior. 11: e1152438. 27074617 10.1080/15592324.2016.1152438 PMC4883921

[nph16887-bib-0035] Gui JS , Zheng S , Shen JH , Li LG . 2015. Grain setting defect1 (GSD1) function in rice depends on S‐acylation and interacts with actin 1 (OsACT1) at its C‐terminal. Frontiers in Plant Science 6: 804. 26483819 10.3389/fpls.2015.00804 PMC4590517

[nph16887-bib-0036] Heilmann I . 2016. Phosphoinositide signaling in plant development. Development 143: 2044–2055. 27302395 10.1242/dev.136432

[nph16887-bib-0037] Heisler MG , Hamant O , Krupinski P , Uyttewaal M , Ohno C , Jönsson H , Traas J , Meyerowitz E . 2010. Alignment between PIN1 polarity and microtubule orientation in the shoot apical meristem reveals a tight coupling between morphogenesis and auxin transport. PLoS Biology 8: e1000516. 20976043 10.1371/journal.pbio.1000516 PMC2957402

[nph16887-bib-0038] Hu HZ , Zhang R , Tao ZS , Li XK , Li YY , Huang JF , Li XX , Han X , Feng SQ , Zhang GM *et al*. 2018. Cellulose synthase mutants distinctively affect cell growth and cell wall integrity for plant biomass production in *Arabidopsis* . Plant and Cell Physiology 59: 1144–1157. 29514326 10.1093/pcp/pcy050

[nph16887-bib-0039] Huang DQ , Sun YB , Ma ZM , Ke MY , Cui Y , Chen ZC , Chen CF , Ji CY , Tran TM , Yang L *et al*. 2019. Salicylic acid‐mediated plasmodesmal closure via Remorin‐dependent lipid organization. Proceedings of the National Academy of Sciences, USA 116: 21274–21284. 10.1073/pnas.1911892116 PMC6800329 31575745

[nph16887-bib-0040] Huang F , Kemel Zago M , Abas L , van Marion A , Galván‐Ampudia CS , Offringa R . 2010. Phosphorylation of conserved PIN motifs directs *Arabidopsis* PIN1 polarity and auxin transport. The Plant Cell 22: 1129–1142. 20407025 10.1105/tpc.109.072678 PMC2879764

[nph16887-bib-0041] Ischebeck T , Werner S , Krishnamoorthy P , Lerche J , Meijón M , Stenzel I , Löfke C , Wiessner T , Im YJ , Perera IY *et al*. 2013. Phosphatidylinositol 4,5‐bisphosphate influences PIN polarization by controlling clathrin‐mediated membrane trafficking in *Arabidopsis* . The Plant Cell 25: 4894–4911. 24326589 10.1105/tpc.113.116582 PMC3903994

[nph16887-bib-0042] Jaillais Y , Fobis‐Loisy I , Miège C , Rollin C , Gaude T . 2006. AtSNX1 defines an endosome for auxin‐carrier trafficking in *Arabidopsis* . Nature 443: 106–109. 16936718 10.1038/nature05046

[nph16887-bib-0043] Jarsch IK , Konrad SSA , Stratil TF , Urbanus SL , Szymanski W , Braun P , Braun KH , Ott T . 2014. Plasma membranes are subcompartmentalized into a plethora of coexisting and diverse microdomains in *Arabidopsis* and *Nicotiana benthamiana* . The Plant Cell 26: 1698–1711. 24714763 10.1105/tpc.114.124446 PMC4036580

[nph16887-bib-0044] Johnson AJ , Gnyliukh N , Kaufmann W , Narasimhan M , Vert G , Bednarek S , Friml J . 2020. Experimental toolbox for quantitative evaluation of clathrin‐mediated endocytosis in the plant model *Arabidopsis* . Journal of Cell Science 133: jcs24806. 10.1242/jcs.248062 32616560

[nph16887-bib-0045] Kalappurakkal JM , Anilkumar AA , Patra CV , Zanten TS , Sheetz MP , Satyajit Mayor . 2019. Integrin mechano‐chemical signaling generates plasma membrane nanodomains that promote cell spreading. Cell 177: 1738–1756. 31104842 10.1016/j.cell.2019.04.037 PMC6879320

[nph16887-bib-0046] Kania U , Fendrych M , Friml J . 2014. Polar delivery in plants; commonalities and differences to animal epithelial cells. Open Biology Journal 4: 140017. 10.1098/rsob.140017 PMC4043115 24740985

[nph16887-bib-0047] Kaufmann WA , Kasugai Y , Ferraguti F , Storm JF . 2010. Two distinct pools of large‐conductance calcium‐activated potassium channels in the somatic plasma membrane of central principal neurons. Neuroscience 169: 974–986. 20595025 10.1016/j.neuroscience.2010.05.070 PMC2923744

[nph16887-bib-0048] Kim I , Kobayashi K , Cho E , Zambryski PC . 2005. Subdomains for transport via plasmodesmata corresponding to the apical–basal axis are established during *Arabidopsis* embryogenesis. Proceedings of the National Academy of Sciences, USA 102: 11945–11950. 10.1073/pnas.0505622102 PMC1188016 16087887

[nph16887-bib-0049] Kleine‐Vehn J , Łangowski L , Wisniewska J , Dhonukshe P , Brewer P , Friml J . 2008. Cellular and molecular requirements for polar PIN targeting and transcytosis in plants. Molecular Plant. 6: 1056–1066. 10.1093/mp/ssn062 19825603

[nph16887-bib-0050] Kleine‐Vehn J , Wabnik K , Martinière A , Łangowski Ł , Willig K , Naramoto S , Leitner J , Tanaka H , Jakobs S , Robert S *et al*. 2011. Recycling, clustering, and endocytosis jointly maintain PIN auxin carrier polarity at the plasma membrane. Molecular Systems Biology 7: 540. 22027551 10.1038/msb.2011.72 PMC3261718

[nph16887-bib-0051] Knepper C , Savory EA , Day B . 2011. *Arabidopsis* NDR1 is an integrin‐like protein with a role in fluid loss and plasma membrane‐cell wall adhesion. Plant Physiology 156: 286–300. 21398259 10.1104/pp.110.169656 PMC3091050

[nph16887-bib-0052] Kusumi A , Sako Y . 1996. Cell surface organization by the membrane skeleton. Current Opinion in Cell Biology. 8: 566–574. 8791449 10.1016/s0955-0674(96)80036-6

[nph16887-bib-0053] Legrand A , Martinez D , Grélard A , Berbon M , Morvan E , Tawani A , Loquet A , Mongrand S , Habenstein B . 2019. Nanodomain clustering of the plant protein remorin by solid‐state NMR. Frontiers in Molecular Biosciences 6: 107. 31681795 10.3389/fmolb.2019.00107 PMC6803476

[nph16887-bib-0054] Leitner J , Petrášek J , Tomanov K , Retzer K , Pařezová M , Korbei B , Bachmair A , Zažímalová E , Luschnig C . 2012. Lysine^63^‐linked ubiquitylation of PIN2 auxin carrier protein governs hormonally controlled adaptation of *Arabidopsis* root growth. Proceedings of the National Academy of Sciences, USA 109: 8322–8327. 10.1073/pnas.1200824109 PMC3361439 22556266

[nph16887-bib-0055] Lewis DR , Muday GK . 2009. Measurement of auxin transport in *Arabidopsis thaliana* . Nature Protocols 4: 437–451. 19282849 10.1038/nprot.2009.1

[nph16887-bib-0056] Li G , Xue H‐W . 2007. *Arabidopsis PLDζ2* regulates vesicle trafficking and is required for auxin response. The Plant Cell 19: 281–295. 17259265 10.1105/tpc.106.041426 PMC1820954

[nph16887-bib-0057] Lin DS , Cao LY , Zhou ZZ , Zhu L , Ehrhardt D , Yang ZB , Fu Y . 2013. Rho GTPase signaling activates microtubule severing to promote microtubule ordering in *Arabidopsis* . Current Biology 23: 290–297. 23394835 10.1016/j.cub.2013.01.022

[nph16887-bib-0058] Liu Z , Persson S , Zhang Y . 2015. The connection of cytoskeletal network with plasma membrane and the cell wall. Journal of Integrative Plant Biology 57: 330–340. 25693826 10.1111/jipb.12342 PMC4405036

[nph16887-bib-0059] Luptovčiak I , Komis G , Takáč T , Ovečka M , Šamaj J . 2017. Katanin: a sword cutting microtubules for cellular, developmental, and physiological purposes. Frontiers in Plant Science 8: 1982. 29209346 10.3389/fpls.2017.01982 PMC5702333

[nph16887-bib-0060] Luschnig C , Vert G . 2014. The dynamics of plant plasma membrane proteins: PINs and beyond. Development 141: 2924–2938. 25053426 10.1242/dev.103424

[nph16887-bib-0061] Lv XQ , Jing YP , Xiao JW , Zhang YD , Zhu YF , Julian R , Lin JX . 2017. Membrane microdomains and the cytoskeleton constrain AtHIR1 dynamics and facilitate the formation of an AtHIR1‐associated immune complex. The Plant Journal 90: 3–16. 28081290 10.1111/tpj.13480

[nph16887-bib-0062] Malinsky J , Opekarova M , Grossmann G , Tanner W . 2013. Membrane microdomains, rafts, and detergent‐resistant membranes in plants and fungi. Annual Review of Plant Biology 64: 501–529. 10.1146/annurev-arplant-050312-120103 23638827

[nph16887-bib-0063] Markham JE , Molino D , Gissot L , Bellec Y , Hematy K , Marion J , Belcram K , Palauqui JC , Satiat‐Jeunemaitre B , Faure JD . 2011. Sphingolipids containing very‐long‐chain fatty acids define a secretory pathway for specific polar plasma membrane protein targeting in *Arabidopsis* . The Plant Cell 23: 2362–2378. 21666002 10.1105/tpc.110.080473 PMC3160045

[nph16887-bib-0064] Martinière A , Lavagi I , Nageswaran G , Rolfe DJ , Maneta‐Peyret L , Luu D‐T , Botchway SW , Webb SED , Mongrand S , Maurel C *et al*. 2012. Cell wall constrains lateral diffusion of plant plasma‐membrane proteins. Proceedings of the National Academy of Sciences, USA 109: 12805–12810. 10.1073/pnas.1202040109 PMC3411962 22689944

[nph16887-bib-0065] Mazur E , Kulik I , Hajný J , Friml J . 2020. Auxin canalization and vascular tissue formation by TIR1/AFB‐mediated auxin signaling in *Arabidopsis* . New Phytologist 226: 1375–1383. 10.1111/nph.16446 PMC7318144 31971254

[nph16887-bib-0066] McKenna JF , Rolfe DJ , Webb SED , Tolmie AF , Botchway SW , Martin‐Fernandez ML , Hawes C , Runions J . 2019. The cell wall regulates dynamics and size of plasma‐membrane nanodomains in *Arabidopsis* . Proceedings of the National Academy of Sciences, USA 116: 12857–12862. 10.1073/pnas.1819077116 PMC6601011 31182605

[nph16887-bib-0067] McKenna JF , Tolmie AF , Runions J . 2014. Across the great divide: the plant cell surface continuum. Current Opinion in Plant Biology 22: 132–140. 25460078 10.1016/j.pbi.2014.11.004

[nph16887-bib-0068] Meijer H , Munnik T . 2003. Phospholipid based signaling in plants. Annual Review of Plant Biology 54: 265–306. 10.1146/annurev.arplant.54.031902.134748 14502992

[nph16887-bib-0069] Mellman I , Nelson WJ . 2008. Coordinated protein sorting, targeting and distribution in polarized cells. Nature Reviews Molecular Cell Biology 9: 833–845. 18946473 10.1038/nrm2525 PMC3369829

[nph16887-bib-0070] Möbius W , Cooper B , Kaufmann WA , Imig C , Ruhwedel T , Snaidero N , Saab AS , Varoqueaux F . 2010. Electron microscopy of the mouse central nervous system. Methods in Cell Biology 96: 475–512. 20869535 10.1016/S0091-679X(10)96020-2

[nph16887-bib-0071] Mockaitis K , Estelle M . 2008. Auxin receptors and plant development: a new signaling paradigm. Annual Review of Cell Developmental Biology 24: 55–80. 10.1146/annurev.cellbio.23.090506.123214 18631113

[nph16887-bib-0072] Mueller‐Roeber B , Pical C . 2002. Inositol phospholipid metabolism in *Arabidopsis*. Characterized and putative isoforms of inositol phospholipid kinase and phosphoinositide‐specific phospholipase C. Plant Physiology 130: 22–46. 12226484 10.1104/pp.004770 PMC166537

[nph16887-bib-0073] Nakayama N , Smith RS , Mandel T , Robinson S , Kimura S , Boudaoud A , Kuhlemeier C . 2012. Mechanical regulation of auxin‐mediated growth. Current Biology 22: 1468–1476. 22818916 10.1016/j.cub.2012.06.050

[nph16887-bib-0074] Narasimhan M , Johnson A , Prizak R , Kaufmann WA , Tan ST , Casillas‐Pérez B , Friml J . 2020. Evolutionarily unique mechanistic framework of clathrin‐mediated endocytosis in plants. eLife 9: e52067. 31971511 10.7554/eLife.52067 PMC7012609

[nph16887-bib-0075] Nelson KS , Beitel GJ . 2009. Cell junctions: lessons from a broken heart. Current Biology 19: R122–R123. 19211050 10.1016/j.cub.2008.12.002

[nph16887-bib-0076] Persson S , Paredez A , Carroll A , Palsdottir H , Doblin M , Poindexter P , Khitrov N , Auer M , Somerville CR . 2007. Genetic evidence for three unique components in primary cell‐wall cellulose synthase complexes in *Arabidopsis* . Proceedings of the National Academy of Sciences, USA 104: 15566–15571. 10.1073/pnas.0706592104 PMC2000526 17878302

[nph16887-bib-0077] Poraty‐Gavra L , Zimmermann P , Haigis S , Bednarek P , Hazak O , Stelmakh OR , Sadot E , Schulze‐Lefert P , Gruissem W , Yalovsky S . 2013. The *Arabidopsis* Rho of plants GTPase AtROP6 functions in developmental and pathogen response pathways. Plant Physiology 161: 1172–1188. 23319551 10.1104/pp.112.213165 PMC3585588

[nph16887-bib-0078] Rakusová H , Abbas M , Han H , Song S , Robert HS , Friml J . 2016. Termination of shoot gravitropic responses by auxin feedback on PIN3 polarity. Current Biology 26: 3026–3032. 27773568 10.1016/j.cub.2016.08.067

[nph16887-bib-0079] Rhee SG , Bae YS . 1997. Regulation of phosphoinositide‐specific phospholipase C isozymes. Journal of Biological Chemistry 272: 15045–15048. 10.1074/jbc.272.24.15045 9182519

[nph16887-bib-0080] Sauer M , Balla J , Luschnig C , Wiśniewska J , Reinöhl V , Friml J , Benková E . 2006a. Canalization of auxin flow by Aux/IAA‐ARF‐dependent feedback regulation of PIN polarity. Genes and Development 20: 2902–2911. 17043314 10.1101/gad.390806 PMC1619939

[nph16887-bib-0081] Sauer M , Paciorek T , Benková E , Friml J . 2006b. Immunocytochemical techniques for whole mount *in situ* protein localization in plants. Nature Protocols. 1: 98–103. 17406218 10.1038/nprot.2006.15

[nph16887-bib-0082] Saunders TE , Pan KZ , Angel A , Guan Y , Shah JV , Howard M , Chang F . 2012. Noise reduction in the intracellular pom1p gradient by a dynamic clustering mechanism. Developmental Cell 22: 558–572. 22342545 10.1016/j.devcel.2012.01.001 PMC3312004

[nph16887-bib-0102] Simon ML , Platre MP , Assil S , van Wijk R , Chen WY , Chory J , Dreux M , Munnik T , Jaillais Y . 2014 A multi‐colour/multi‐affinity marker set to visualize phosphoinositide dynamics in Arabidopsis. Plant Journal 77: 322–337. 10.1111/tpj.12358 PMC3981938 24147788

[nph16887-bib-0083] Shoji T , Narita NN , Hayashi K , Asada J , Hamada T , Sonobe S , Nakajima K , Hashimoto T . 2004. Plant‐specific microtubule‐associated protein SPIRAL2 is required for anisotropic growth in *Arabidopsis* . Plant Physiology 136: 39334–3944. 10.1104/pp.104.051748 PMC535826 15557095

[nph16887-bib-0084] Skokan R , Medvecká E , Viaene T , Vosolsobě S , Zwiewka M , Müller K , Skůpa P , Karady M , Zhang YZ , Janacek DP *et al*. 2019. PIN‐driven auxin transport emerged early in streptophyte evolution. Nature Plants 5: 1114–1119. 31712756 10.1038/s41477-019-0542-5

[nph16887-bib-0085] Skotland T , Sandvig K . 2019. The role of PS 18:0/18:1 in membrane function. Nature Communications. 10: 2752. 10.1038/s41467-019-10711-1 PMC6588574 31227693

[nph16887-bib-0086] Stone BA , Evans NA , Bonig I , Clarke AE . 1984. The application of sirofluor, a chemically defined fluorochrome from aniline blue for the histochemical detection of callose. Protoplasma 122: 191–195.

[nph16887-bib-0088] Tan ST , Abas M , Verstraeten I , Glanc M , Molnár G , Hajný J , Lasák P , Petřík I , Russinova E , Petrášek J *et al*. 2020a. Salicylic acid targets protein phosphatase 2A to attenuate growth in plants. Current Biology 30: 381–395. 31956021 10.1016/j.cub.2019.11.058 PMC6997888

[nph16887-bib-0089] Tan ST , Zhang X , Kong W , Yang XL , Molnár G , Vondráková Z , Filepová R , Petrášek J , Friml J , Xue HW . 2020b. The lipid code‐dependent phosphoswitch PDK1–D6PK activates PIN‐mediated auxin efflux in *Arabidopsis* . Nature Plants 6: 556–559. 32393881 10.1038/s41477-020-0648-9

[nph16887-bib-0090] Tapken W , Murphy AS . 2015. Membrane nanodomains in plants: capturing form function and movement. Journal of Experimental Botany 66: 1573–1586. 25725094 10.1093/jxb/erv054

[nph16887-bib-0091] Tejos R , Sauer M , Vanneste S , Palacios‐Gomez M , Li H , Heilmann M , van Wijk R , Vermeer JEM , Heilmann I , Munnik T *et al*. 2014. Bipolar plasma membrane distribution of phosphoinositides and their requirement for auxin‐mediated cell polarity and patterning in *Arabidopsis* . The Plant Cell 26: 2114–2128. 24876254 10.1105/tpc.114.126185 PMC4079372

[nph16887-bib-0092] Wightman R , Chomicki G , Kumar M , Carr P , Turner SR . 2013. SPIRAL2 determines plant microtubule organization by modulating microtubule severing. Current Biology 23: 1–6. 24055158 10.1016/j.cub.2013.07.061 PMC3793865

[nph16887-bib-0093] Wiśniewska J , Xu J , Seifertová D , Brewer PB , Růžička K , Blilou I , Rouquié D , Benková E , Scheres B , Friml J . 2006. Polar PIN localization directs auxin flow in plants. Science 312: 883. 16601151 10.1126/science.1121356

[nph16887-bib-0094] Wolf S , Hématy K , Höfte H . 2012a. Growth control and cell wall signaling in plants. Annual Review of Plant Biology 63: 381–407. 10.1146/annurev-arplant-042811-105449 22224451

[nph16887-bib-0095] Wolf S , Mravec J , Greiner S , Mouille G , Höfte H . 2012b. Plant cell wall homeostasis is mediated by brassinosteroid feedback signaling. Current Biology 22: 1732–1737. 22885061 10.1016/j.cub.2012.07.036

[nph16887-bib-0096] Xue HW , Chen X , Mei Y . 2009. Function and regulation of phospholipid signalling in plants. Biochemical Journal 421: 145–156. 10.1042/BJ20090300 PMC2708932 19552624

[nph16887-bib-0097] Yang Z‐B , Geng X , He C , Zhang F , Wang R , Horst WJ , Ding Z . 2014. TAA1‐regulated local auxin biosynthesis in the root‐apex transition zone mediates the aluminum‐induced inhibition of root growth in *Arabidopsis* . The Plant Cell 26: 2889–904. 25052716 10.1105/tpc.114.127993 PMC4145121

[nph16887-bib-0098] Zhang J , Nodzyński T , Pĕnčík A , Rolčík J , Friml J . 2010. PIN phosphorylation is sufficient to mediate PIN polarity and direct auxin transport. Proceedings of the National Academy of Sciences, USA 107: 918–922. 10.1073/pnas.0909460107 PMC2818920 20080776

[nph16887-bib-0099] Zhang X , Adamowski M , Marhava P , Tan ST , Zhang YZ , Rodriguez L , Zwiewka M , Pukyšová V , Sánchez AS , Raxwal VK *et al*. 2020. *Arabidopsis* flippases cooperate with ARF GTPase exchange factors to regulate the trafficking and polarity of PIN auxin transporters. The Plant Cell 32: 1644–1664. 32193204 10.1105/tpc.19.00869 PMC7203944

[nph16887-bib-0100] Zwiewka M , Nodzyński T , Robert S , Vanneste S , Friml J . 2015. Osmotic stress modulates the balance between exocytosis and clathrin‐mediated endocytosis in *Arabidopsis thaliana* . Molecular Plant 8: 1175–1187. 25795554 10.1016/j.molp.2015.03.007

