# Supplementary material for: Cellular requirements for PIN polar cargo clustering in Arabidopsis thaliana
Source: New Phytol. 2020 Sep 18;229(1):351–69. doi: 10.1111/nph.16887 (PMC7984064; doi:10.1111/nph.16887)
Supplement: Supplementary file 1 — Fig. S1 Visualization and quantification of PIN clusters by confocal microscopy in different zones of the root tip. Fig. S2 Quantification of the polar cargo distribution in different polar domains from IEM images. Fig. S3 Specific PIN2 localization to epidermal and cortex cells confirmed by SDS‐FRL. Fig. S4 Independent PIN cluster positions from several PM structures. Fig. S5 Clustering after treatment with AlCl3 or latrunculin. Fig. S6 No effect of cell‐wall‐digesting chemical on PIP2a‐GFP compared to PIN2‐Venus. Fig. S7 PIN2‐Venus clustering after plasmolysis by IEM. Fig. S8 Increased auxin transport in hypocotyls of spr2‐2, pip5k1 pip5k2 and ndr1 mutants. Methods S1 SDS‐digested freeze‐fracture replica labeling. Table S1 Manual quantification of PIN2 clusters in the pip5k1 pip5k2 mutant (Figure 3). [file NPH-229-351-s001.pdf]

***New Phytologist* Supporting Information**

Article acceptance date: 7 August 2020

Article title: Cellular requirements for PIN polar cargo clustering in *Arabidopsis thaliana*
Authors: Hongjiang Li, Daniel von Wangenheim, Xixi Zhang, Shutang Tan, Nasser Darwish-Miranda, Satoshi Naramoto, Krzysztof Wabnik, Riet De Rycke, Walter A. Kaufmann, Daniel Gütl, Ricardo Tejos, Peter Grones, Meiyu Ke, Xu Chen, Jan Dettmer and Jiří Friml

The following Supporting Information is available for this article:

Fig. S1 Visualization and quantification of PIN clusters by confocal microscopy in different zones of the root tip.

Fig. S2 Quantification of the polar cargo distribution in different polar domains from IEM images.

Fig. S3 Specific PIN2 localization to epidermal and cortex cells confirmed by SDS-FRL.

Fig. S4 Independent PIN cluster positions from several PM structures.

Fig. S5 Clustering after treatment with $AlCl_3$ or Latrunculin.

Fig. S6 No effect of cell wall digesting chemical on PIP2a-GFP compared to PIN2-Venus.

Fig. S7 *PIN2-Venus* clustering after plasmolysis by IEM.

Fig. S8 Increased auxin transport in hypocotyls of *spr2-2*, *pip5k1 pip5k2* and *ndr1* mutants.

Table S1 Manual quantification of PIN2 clusters in the *pip5k1 pip5k2* mutant (Figure 3).

Methods S1 SDS-digested freeze-fracture replica labeling

Video S1 Three-dimensional rotation of *PIN2-GFP* clusters.

Video S2 *PIN2-GFP* root cap and epidermal cell clusters during root growth.

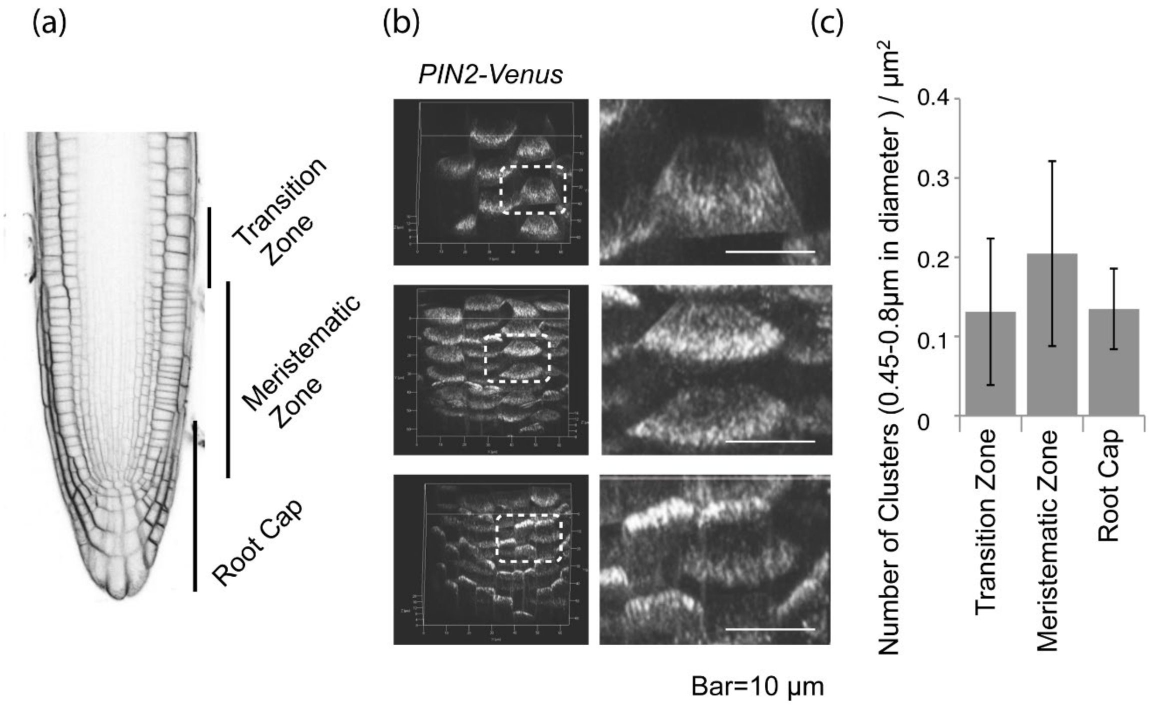

Fig. S1 Visualization and quantification of PIN clusters by confocal microscopy in different zones of the root tip. (a) Schematic representation of different root tip zones. (b) Three-dimensional view of PIN2-Venus clustering at the transition zone (top), meristematic zone (middle), and root cap (bottom). The right panels are enlargements of the boxed areas in the left panels. Bars = 10 µm. (c) Quantitative analysis of the cluster density in (b). Values are means ± SD.

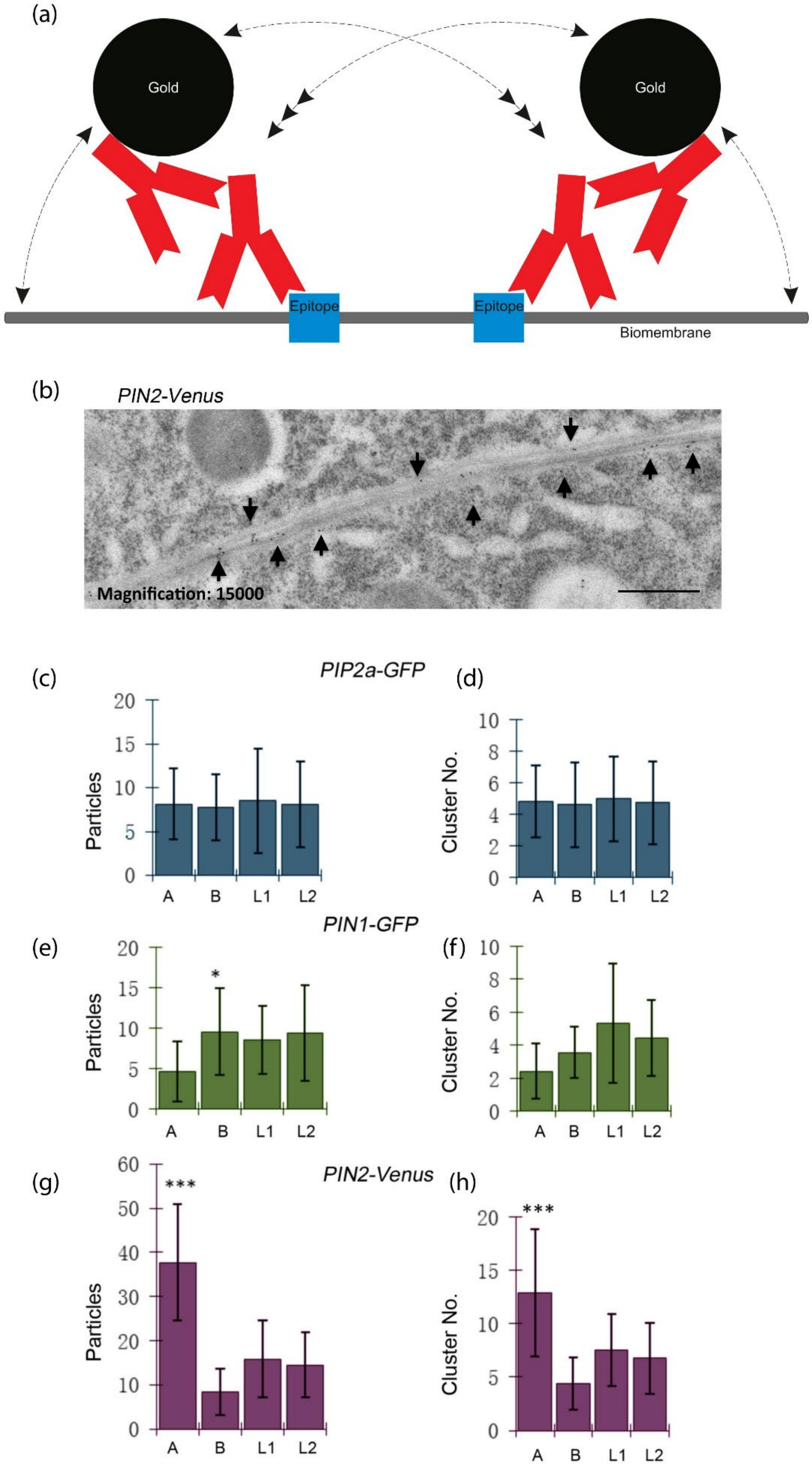

(a)
Gold
Gold
Epitope
Epitope
Biomembrane
(b)
PIN2-Venus
Magnification: 15000
(c)
PIP2a-GFP
(d)
Particles
Cluster No.
A
B
L1
L2
(e)
PIN1-GFP
(f)
(g)
PIN2-Venus
(h)

Fig. S2 Quantification of the polar cargo distribution in different polar domains from IEM images. (a) Schematic representation of the cluster definition in IEM. The center of the gold particles can be as far as 20 nm from the corresponding epitopes because of the size of the interspersed immunoglobulins (approximately 8 nm; red), the size of the particles themselves (10 nm; black), and free rotation around the epitopes. Estimated size of the PIN2 epitope is 2.5 nm (blue). Most stable dimer association in biomembranes has been reported to be 10 nm (Casuso *et al.*, 2010). One label can be detected per protein when using 5-nm or even larger gold particle sizes because of steric hindrances. Using monoclonal or polyclonal antibodies does not make any difference with respect to clustering in this case. Hence, protein interaction and cluster formation occurred when the distance between neighboring gold particles was less than 55 nm. The "maximum" distance of a particle to its epitope and the distance of proteins when they are still interacting have been shown. Naturally, gold particles can be found anywhere within this range, even closer together as the actual epitopes. Homogeneously dispersed epitopes will always give a homogeneous pattern and non-homogeneously dispersed epitopes a non-homogeneous pattern. (b) At a 15,000 magnification, assignment of *PIN2-Venus* clusters in IEM to different domains based on their position relative to the cell wall and plasma membrane indicated by the arrow directions. Arrows pointing up and down represent apically and basally localized clusters, respectively. Bar = 1 µm. (c) to (h) Quantitative analysis of IEM cluster images. Average number of particles and cluster numbers in *PIP2a-GFP* (c, d), *PIN1-GFP* (e, f), and *PIN2-Venus* (g and h) at different polar domains; apical (a), basal (b), and lateral (L1 and L2). Values are means ± SD. Mann-Whitney U test *$P < 0.1$; *** $P < 0.001$.

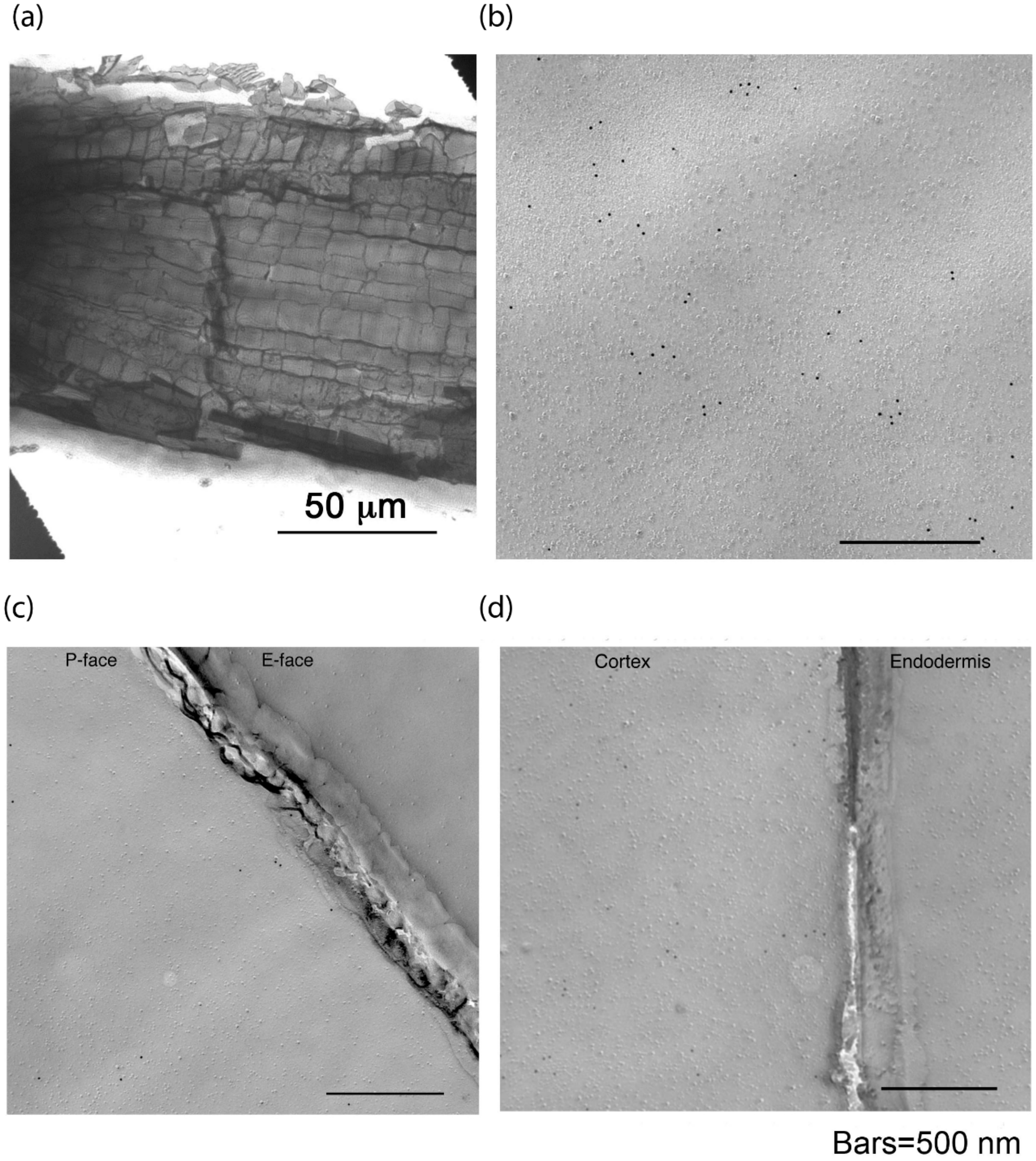

Fig. S3 Specific PIN2 localization to epidermal and cortex cells confirmed by SDS-FRL. (a) Overview picture of a root tip fractured and replicated in the longitudinal plane. Bar = 50 µm. (b) Non-homogeneous distribution of immunogold particles labeling PIN2 in the plasma membrane of epidermal cells forming loose aggregations and tight clusters. (c) Restricted immunolabeling to the plasma membrane leaflet facing the protoplasmic site (P-face of the membrane) with more bumps (proteins) anchored on cytoplasmic, confirming the epitope localization at the intracellular side. The E-face membrane

(leaflet facing the extracellular space) is free of any immunolabeling. (d) Specific PIN2 localization in the endodermis and cortex. Endodermis cells are free of any immunolabeling. Bars = 500 nm in (b) to (d).

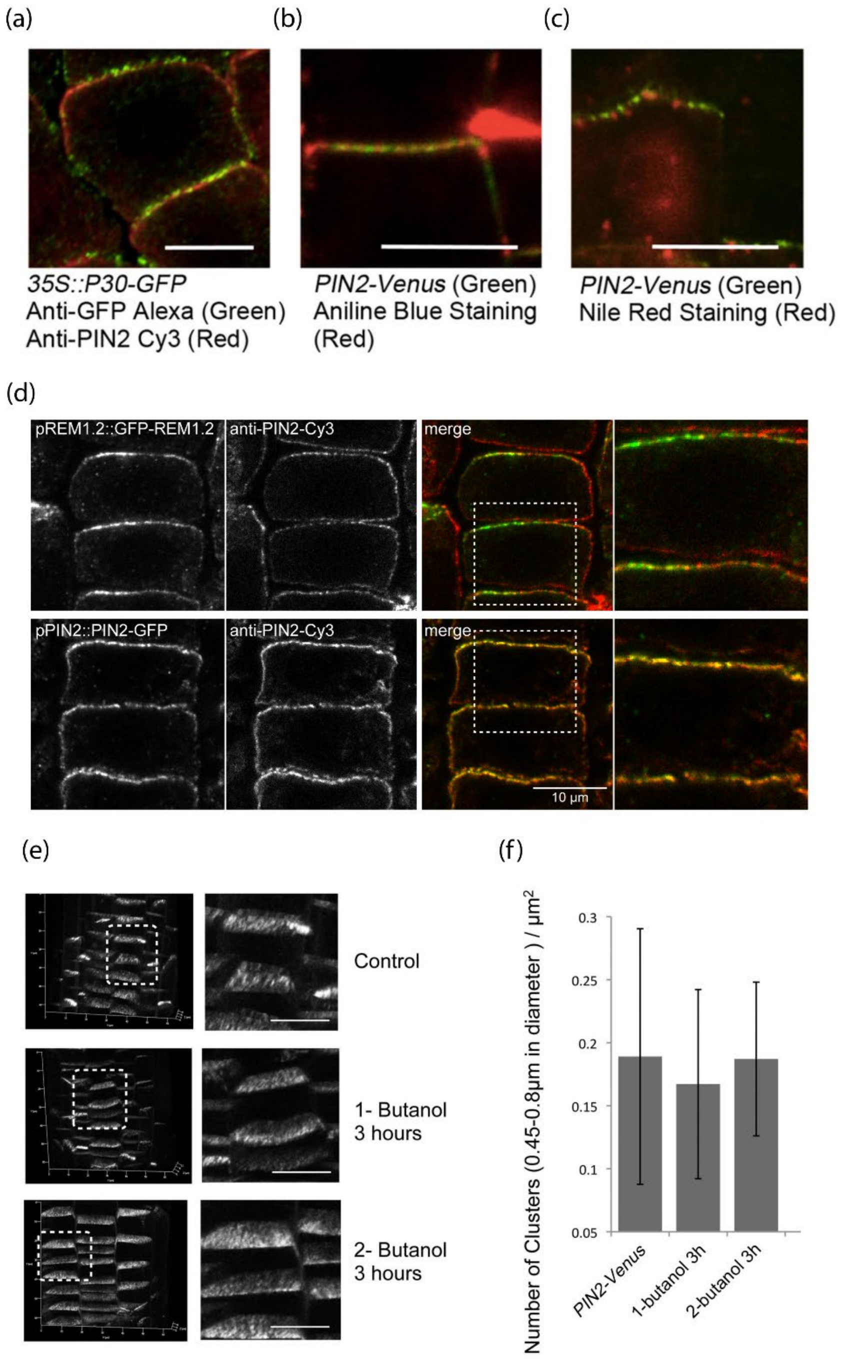

Fig. S4 Independent PIN cluster positions from several PM structures. (a) Immunocolocalization of PIN2-Venus clusters (red) with plasmodesmata marker *35S::P30-GFP* (green). Plasmodesmata were immunostained with anti-GFP primary and Alexa-conjugated secondary antibodies and PIN2 with anti-PIN2 primary and Cy3-conjugated secondary antibodies. (b) Colocalization of PIN2-Venus clusters (green) with

callose assayed by aniline blue staining (red). (c) Colocalization of PIN2-Venus clusters (green) with lipid rafts assayed by Nile red staining (red). (d) Plants expressing *REM1.2::GFP-REM1.2* (upper panel) and control plants expressing *PIN2::PIN2-GFP* (lower panel) were immunostained with anti-PIN2 primary and Cy3-conjugated secondary antibodies. (e) Three-dimensional view of PIN2-Venus clustering after 0.8% (v/v) 1-butanol (phospholipase D inhibitor) and 2-butanol (analog of 1-butanol) treatments. The boxed area is enlarged in the image panel on the right. (f) Quantitative analysis of the cluster density in (e). Values are means ± SD. All Bars = 10 µm.

(a)

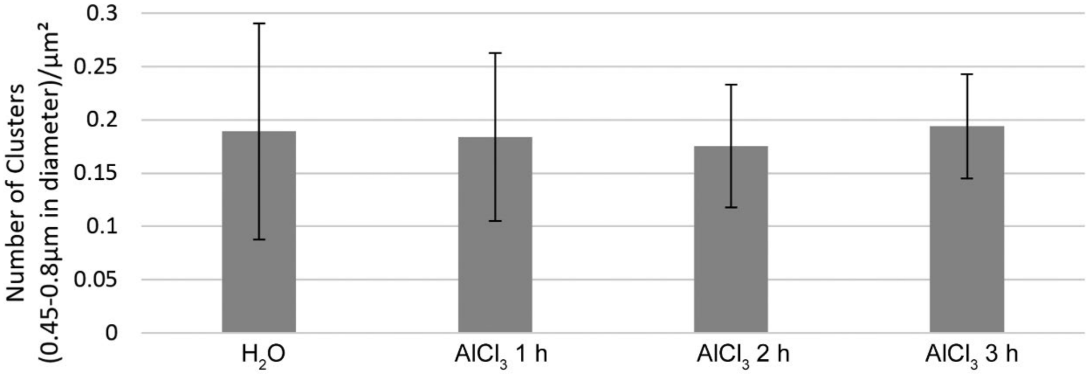

(b)

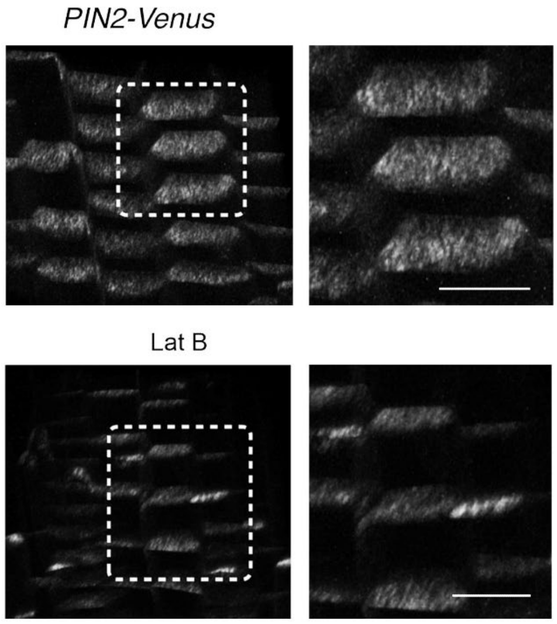

(c)

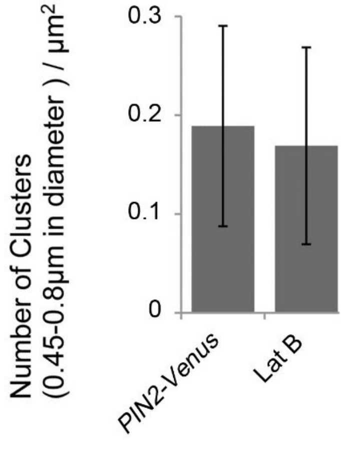

Fig. S5 Clustering after treatment with $AlCl_3$ or Latrunculin. (a) Number of clusters quantified from confocal images of PIN2-Venus plants treated with 100μM $AlCl_3$. Values are means ± SD. (b) Three-dimensional view of clustering in untreated *PIN2-Venus* and after treatment with microfilament organization disruptor latrunculin B (Lat B). Boxed regions are enlarged in the right panels. Bars = 10 μm. (c) Quantitative analysis of the cluster density in (b). Values are means ± SD.

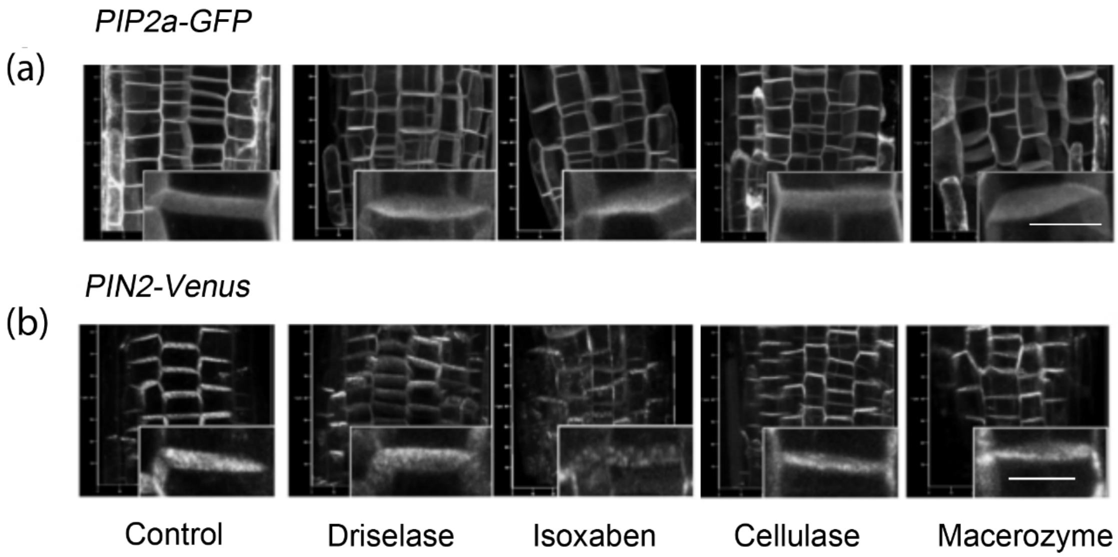

Fig. S6 Decreased clustering by cell wall component breakdown. (a) Unaffected membrane signal of PIP2a-GFP after breakdown of cell walls by driselase, isoxaben, cellulase, and macerozyme treatments, but reduced clustering of PIN2-Venus (b). The signal intensity of the images was adjusted to the same level for easier comparison and membrane regions were enlarged (inset). Bars = 10 μm.

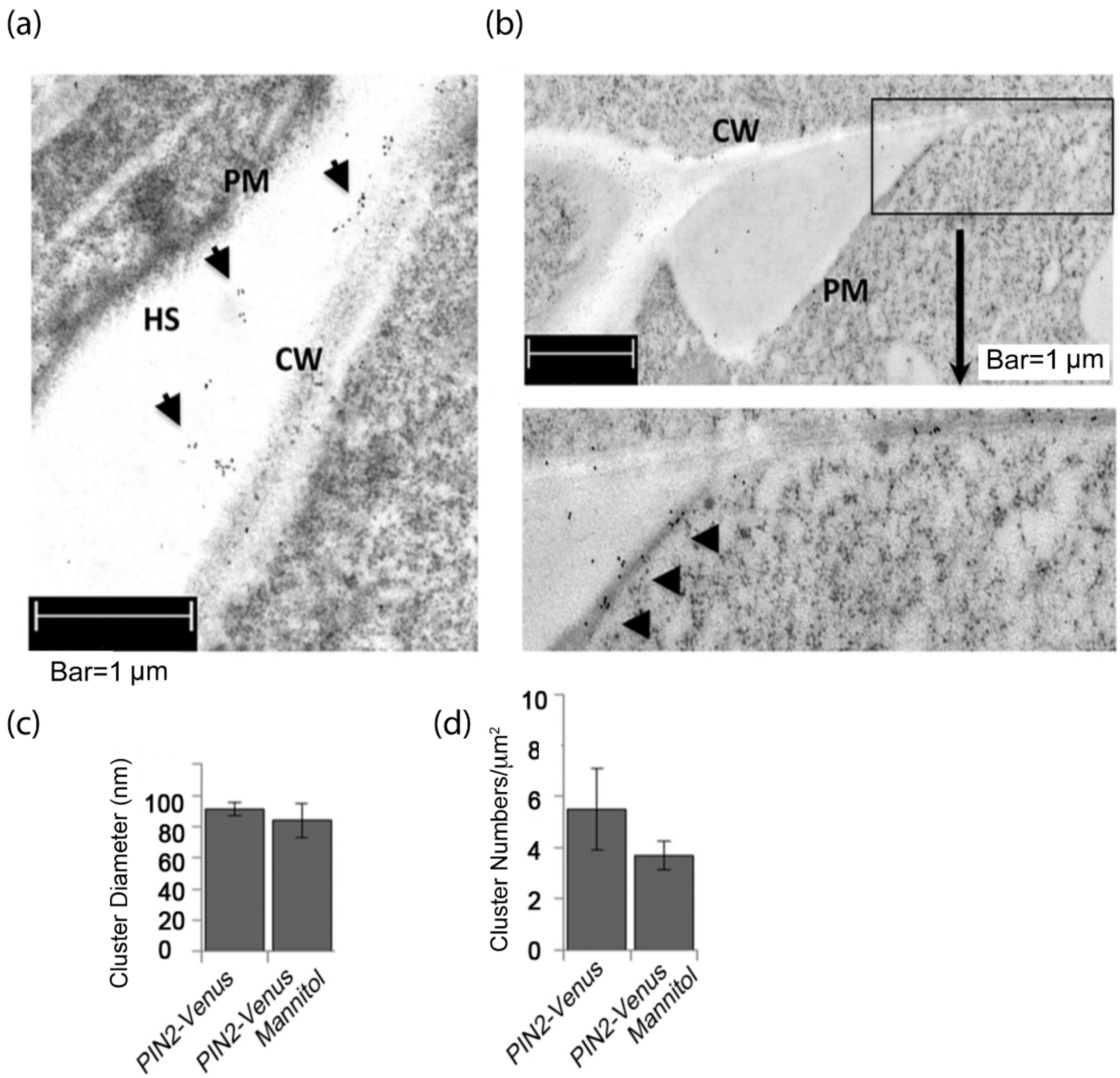

Fig. S7 *PIN2-Venus* clustering after plasmolysis by IEM. (a) Clusters of *PIN2-Venus* (arrows) on hechtian strands (HS). CW, cell wall; PM, plasma membrane. Bar = 1 µm. (b) Clusters of *PIN2-Venus* on the PM after plasmolysis and enlargement of the boxed area. Arrowheads point to the clusters. Bar = 1 µm. (c, d) Quantitative analysis of the cluster diameters (c) and density (d) after plasmolysis by mannitol treatment. Values are means ± SD.

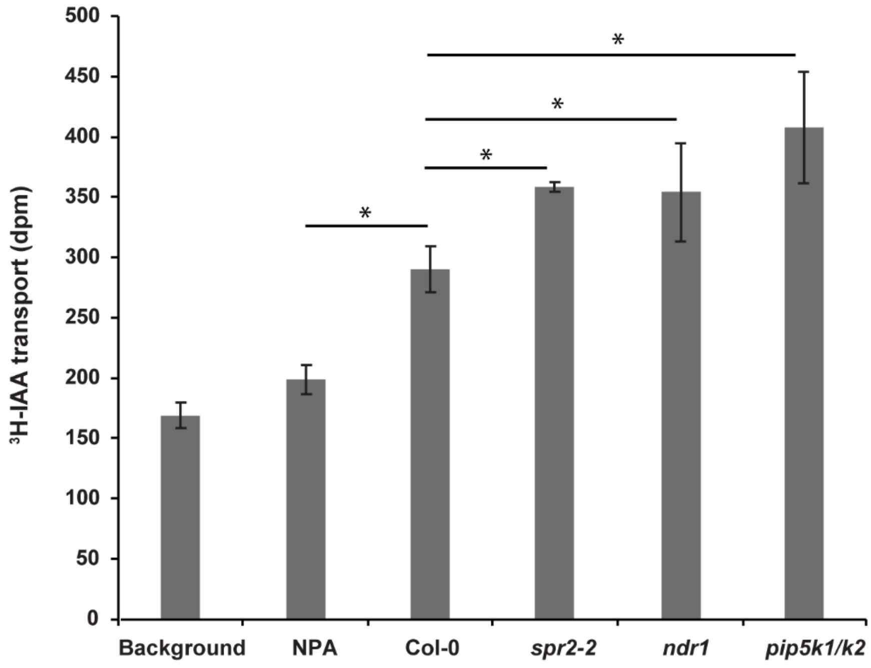

Fig. S8 Increased auxin transport in hypocotyls of *spr2-2*, *pip5k1 pip5k2* and *ndr1* mutants. Intact etiolated hypocotyls of 6-d-old Col-0, *spr2-2*, *pip5k1 pip5k2* and *ndr1* seedlings were used to measure the ^{3}H-IAA transport. For each genotype, 15 hypocotyls were pooled as one replicate, with 3 replicates. Background indicates the sample only with the scintillation solution. N-1-naphthylphthalamic acid (NPA) is an inhibitor of auxin transport. 5 μM NPA treatment was used as a control for inhibition of auxin transport. The 6-d-old etiolated Col-0 seedlings were transferred to the MS medium plate supplemented with 5 μM NPA, droplets of agarose containing ^{3}H-IAA were placed to the shootward end of decapitated etiolated Col-0 seedlings. Values are means ± SD. *t*-test, * $P < 0.05$.

Table S1 Manual quantification of PIN2 clusters in the *pip5k1 pip5k2* mutant (Fig.3).

Experimental data[a]	Number of clusters (n)		Apical cell side width (μm)		Cluster density (n/μm)	
	Wild type	*pip5k1 pip5k2*	Wild type	*pip5k1 pip5k2*	Wild type	*pip5k1 pip5k2*
1	13.56	8.33	13.56	13.68	1.00	0.62
2	14.08	10.22	14.39	13.1	0.99	0.77
3	11.76	10.00	14.46	14.74	0.81	0.64
Average	13.13	9.52	14.14	13.84	0.94	0.68
SD	1.22	1.03	0.5	0.83	0.11	0.08
SE	0.70	0.60	0.29	0.48	0.06	0.05
P^b		0.0172**		0.6246		0.0297**

[a] Three biological replicates per experiment; [b] unpaired two-tailed Student's *t*-test. ** $P < 0.05$.

Methods S1 SDS-digested freeze-fracture replica labeling

Root tips of 3-day-old seedlings of wild-type and transgenic *Arabidopsis* lines were excised, immersed in 5% (w/v) sucrose in water, and frozen immediately by a high-pressure freezing machine (HPM 010; Bal-Tec). Samples were fractured by a double replica method (tensile fracture) in a freeze-etching device (BAF 060; Bal-Tec). Fractured faces were replicated by evaporation of carbon (rotating) by means of an electron beam gun positioned at a 90° angle to a thickness of 5 nm and shadowed unidirectional with platinum-carbon at a 60° angle (thickness 2 nm). Finally, a 20-nm thick layer of carbon was applied from a 90° angle (rotating). After replication, the cell wall was degraded by application of 2% (w/v) Driselase® (Sigma-Aldrich) in Tris-buffered saline (TBS; 50 mM Tris-HCl, 0.9% [w/v] NaCl, pH 7.4) on a shaking platform for 30 min at 37°C.

Nonreplicated tissue was solubilized in a solution containing 2.5% (w/v) sodium dodecyl sulfate (SDS) and 20% (w/v) sucrose made up in 15 mM TBS buffer, pH 8.3, on a shaking platform for 18 h at 80°C. Replicas were kept in the same solution at room temperature until processed further.

For immunolabeling, replicas were washed in TBS containing 0.05% (w/v) BSA and incubated in a blocking solution containing 5% (w/v) BSA and 1.5% (w/v) FSG in TBS for 1 h at room temperature. Subsequently, replicas were incubated in primary antibody diluted in TBS containing 2% (w/v) BSA overnight at 6°C. We used both antibodies against GFP (indirect labelling) and against PIN2 (direct labelling); both produced similar results. Dilution of antibodies used was: anti-GFP rabbit polyclonal (ab6556; Abcam) at 1:500; anti-GFP mouse monoclonal (031M4761; Sigma-Aldrich) at 1:500; and anti-PIN2 rabbit polyclonal (Abas *et al.*, 2006) at 1:2500. After several washes in TBS, the replicas were reacted with gold-conjugated IgG secondary antibodies (British BioCell International) at 1:30 in 2% (w/v) BSA-containing TBS at 6°C overnight. They were washed in MilliQ water, mounted on formvar-coated 100-line copper grids, and analyzed in a transmission electron microscope (Tecnai 12; FEI) operated at 120 kV and equipped with a charge-coupled device camera (OSIS Veleta; Soft Imaging Systems). Whole images were level adjusted, sharpened, and cropped in Photoshop (Adobe) without changing any specific features within.

References

Casuso I, Sens P, Rico F, Scheuring S. 2010. Experimental evidence for membrane-mediated protein-protein interaction. *Biophysical Journal* **99**: L47–9.

Abas L, Benjamins R, Malenica N, Paciorek T, Wiśniewska J, Moulinier-Anzola JC, Sieberer T, Friml J, Luschnig C. 2006. Intracellular trafficking and proteolysis of the *Arabidopsis* auxin-efflux facilitator PIN2 are involved in root gravitropism. *Nature Cell Biology* **8**: 249-256.
